# Deciphering the Anti‐Diabetic Potential of *Gymnema Sylvestre* Using Integrated Computer‐Aided Drug Design and Network Pharmacology

**DOI:** 10.1111/jcmm.70349

**Published:** 2025-01-14

**Authors:** Amal Mayyas, Ali Al‐Samydai, Amjad Ibrahim Oraibi, Nawres Debbabi, Sara S. Hassan, Hany Aqeel Al‐Hussainy, Ahmad Mohammad Salamatullah, Musaab Dauelbait, Mohammed Bourhia, Khalid S. Almaary

**Affiliations:** ^1^ Faculty of Health Sciences, Department of Pharmacy American University of Madaba Madaba Jordan; ^2^ Pharmacological and Diagnostic Research Centre (PDRC), Faculty of Pharmacy Al‐Ahliyya Amman University Amman Jordan; ^3^ Department of Pharmacy AL‐Manara College for Medical Sciences Amarah Iraq; ^4^ Research Laboratory for Bioactive Natural Products and Biotechnology LR24ES14, Faculty of Dental Medicine of Monastir University of Monastir Monastir Tunisia; ^5^ Department of Pharmacy Hilla University College Babylon Iraq; ^6^ Al‐Nisour University College, Pharmacy Department Baghdad Iraq; ^7^ Department of Food Science & Nutrition, College of Food and Agricultural Sciences King Saud University Riyadh Saudi Arabia; ^8^ Department of Scientific Translation, Faculty of Translation University of Bahri Khartoum Sudan; ^9^ Swalife Biotech Ltd Unit 3D North Point House North Point Business Park Cork Ireland; ^10^ Department of Botany and Microbiology, College of Science King Saud University Riyadh Saudi Arabia

**Keywords:** diabetes, *Gymnema Sylvestre*, molecular docking, molecular dynamics, network pharmacology

## Abstract

This study explores novel therapeutic avenues for diabetes, a global health concern marked by elevated blood glucose levels. We investigated the anti‐diabetic potential of *Gymnema Sylvestre's* bioactive compounds, including Gymnemic acid I, Stigmasterol, Deacylgymnemic acid, Beta‐Amyrin acetate, Longispinogenin, Gymnemic acid II, Gymnemic acid, Gymnemic acid X, Gymnemaside VI, Phytic acid and Gymnemic acid X. Employing network pharmacology, molecular docking and molecular dynamics (MD), we elucidated the potential mechanism of action. SwissTargetPrediction identified targets for bioactive constituents, while DisGeNET provided diabetes‐related targets. A GeneVenn diagram revealed 397 common potential targets for diabetes management. The protein–protein interaction network, constructed via the STRING database, underwent topological analysis in Cytoscape, identifying AKT1, SRC, TNF, PPARG and IL1B as top targets. Gene ontology analysis using FunRich identified crucial roles of screened targets in integrin family cell surface interactions and glypican pathways for diabetes management. Molecular interactions and binding affinities with the top target, AKT1, were assessed, with Gymnemic acid I displaying the least binding energy (−9.813) with H‐ and non‐H‐bond interactions. Molecular dynamics simulations provided insights into the distinct behaviours of Gymnemic acid I within the protein complex. In conclusion, our study elucidates the potential anti‐diabetic mechanism of Gymnemic acid I, underscoring the need for further in vitro, in vivo and clinical studies to validate our findings.

## 1 Introduction

Using natural and holistic remedies for treating diseases has long been a focus of interest, dating back to ancient practices and continuing today as naturopathic medicine gains more attention. India's rich biodiversity includes approximately 45,000 plant species, many of which have been used in traditional medicine systems. Extensive research has explored identifying bioactive compounds from these thousands of Indian medicinal plants that may have beneficial pharmacological properties for drug development or treating illnesses [1, 2]. Diabetes mellitus is a persistent condition marked by an insufficient production of insulin, either relative or absolute, leading to elevated blood sugar levels known as hyperglycemia. In its most severe forms, diabetes can cause multi‐organ complications, including retinopathy, neuropathy, nephropathy and elevated susceptibility to cardiovascular disease, by disrupting the normal function of several bodily systems [3, 4]. While conventional drug treatments like sulfonylureas and biguanides are used, their adverse side effects present a major limitation. As a result, interest has grown in using herbal medicines as potentially safer and more effective alternatives for managing diabetes. Many plants have shown promise for anti‐diabetic properties, making them valuable subjects of ethnobotanical research. Bioactive compounds may be found in various parts of these plants that exhibit differing degrees of blood sugar lowering and antihyperglycemic effects [2, 5]. The isolation of these natural compounds could provide new drug discoveries, potential lead molecules for drug development, or potential pharmacological agents. On the basis of the results of previous animal studies and human clinical trials, certain herbal medicines have demonstrated potential as treatment options for diabetes mellitus. In addition to *Trigonella foenum graecum*, *Gymnema sylvestre* , *Tinospora cordifolia*, *Momordica charantia* and *Curcuma longa* have also been found to be effective against diabetes [6, 7, 8]. This plant extract, fraction or isolated compound has been shown to have the ability to reduce blood glucose levels and improve metabolic abnormalities associated with diabetes in both animal models and humans [9].

Researchers have investigated the safety and efficacy of medicinal plants, thereby revealing the therapeutic potential inherent in traditional medicinal practices. The chemical compounds contained in medicinal plants are renowned for their therapeutic properties. A good example of such a medicinal plant is *Gymnema Sylvestre* , which has long been used in traditional medicine and nicknamed ‘sugar destroyer’ to emphasise its potential therapeutic effects on diabetes. A variety of phytochemicals, including gymnemic acids, flavonoids, saponins and alkaloids, are present in this herb, contributing to its bioactivity (asthma, diabetes, hypercholesterolemia, osteoporosis, microorganisms, diuretic, anaemia, cardiopathy, constipation and indigestion) [10, 11, 12]. In research conducted on *Gymnema Sylvestre* , its anti‐diabetic properties and mechanisms of action have been discovered. Studies suggest it may increase insulin sensitivity, help regenerate pancreatic beta cells responsible for insulin production, and decrease intestinal glucose absorption from food. The demonstrated efficacy in lowering blood sugar levels positions *Gymnema Sylvestre* as a promising botanical treatment for managing and controlling diabetes [13, 14]. Multiple compounds identified in *Gymnema Sylvestre* contribute to its therapeutic potential, particularly for diabetes management. Gymnemic Acid I demonstrates anti‐diabetic effects by inhibiting intestinal glucose absorption and regulating blood sugar levels [15]. Stigmasterol, Longispinogenin, Phytic Acid, Beta‐Amyrin Acetate and Deacylgymnemic Acid exhibit anti‐inflammatory and antioxidant properties and are being studied for diabetes and cholesterol regulation [16, 17, 18, 19]. Variations such as Gymnemic Acid II, IV and X also reduce sugar absorption. Gymnemaside VI, a saponin, is under study for pharmacological activities like anti‐diabetic effects [2]. These essential compounds provide multiple bioactivities that underlie *Gymnema Sylvestre's* use in traditional medicine, particularly for diabetes management. However, additional research is warranted to comprehensively elucidate the clinical efficacy and safety of *Gymnema sylvestre* in human subjects. Therefore, it is imperative to thoroughly assess the anti‐diabetic mechanism to discern the specific effects of *Gymnema Sylvestre* in treating diabetes.

Network pharmacology, an emerging discipline, leverages systems biology methodologies to analyse biological networks and develop drugs capable of modulating multiple targets [20]. This comprehensive understanding at the systems level aids in elucidating the mechanisms of formulations, particularly in terms of synergistic actions on specific nodes within biological networks. Researchers used network pharmacology to uncover pathways and test medicinal plants for diabetic treatment. Molecular docking, a computer method, was utilised to analyse drug‐protein interactions and assess binding strengths. Li et al. (2023) studied ginseng for diabetes management [21], Fu et al. (2023) investigated the molecular mechanism of rhubarb for treating diabetic nephropathy [22], and Arif et al. (2021) identified the molecular mechanism of *Momordica charantia* for treating type 2 diabetes mellitus using network pharmacology and molecular docking methods [23].

To better understand the mechanisms of action, safety and potential efficacy of *Gymnema Sylvestre* bioactive compounds in diabetes management, this study employs an integrative approach. Network pharmacology methods were used to identify relevant targets, diseases and biological pathways (BPA) associated with diabetes. Molecular docking studies examined binding affinities between bioactive compounds and proteins, while molecular dynamics (MD) simulations allowed us to observe the stability and conformational changes in compound‐protein complexes. Together, these approaches provide preliminary insights into molecular interactions and pathways relevant to *Gymnema sylvestre's* potential anti‐diabetic effects.

## 2 Materials and Methods

In this study, we curated all bioactive molecules from *Gymnema Sylvestre* to evaluate its therapeutic potential in diabetes management comprehensively. This approach allowed us to consider a broad range of compounds beyond well‐known constituents to explore various bioactive properties that may influence diabetes‐related pathways.

### 2.1 Data Collection and Retrieval

#### 2.1.1 Proteins Associated With the Development of Diabetes

The proteins involved in the progression of diabetes were identified from the DisGeNET database (https://www.disgenet.org/), the largest database of human disease targets and proteins [24].

#### 2.1.2 Identification of Drug‐Target Information

The active compounds of ***Gymnema Sylvestre*** screened by Indian Medicinal Plants, Phytochemistry Additionally, Therapeutics (IMPPAT) (https://cb.imsc.res.in/imppat/) [25] and KNApSAcK (http://knapsack3d.sakura.ne.jp/) [26] and additional literature search was carried out using Google scholar and PubChem with ‘ *Gymnema Sylvestre* ’ keyword. The compound targets were identified using SwissTargetPrediction (http://swisstargetprediction.ch/) [27].

#### 2.1.3 Identification of Common Targets

GeneVenn diagram was constructed (https://www.bioinformatics.org/gvenn/) [28] to identify the common targets of active constituents of ***Gymnema Sylvestre*** and diabetes.

#### 2.1.4 Protein Interaction (PPI) Network by GeneMANIA

STRING (https://string‐db.org/), a web‐based database, was used to visualise the protein–protein interaction of the common compound‐disease targets [29]. The common targets identified using the GeneVenn diagram were entered in the search box, and the PPI network was constructed after selecting an organism such as *Homo sapiens*. The network was then exported as a .tsv file for further analysis.

### 2.2 Network Analysis

The .tsv file of the PPI (Protein–Protein Interaction) network from the STRING database was imported into Cytoscape v3.9.1 software for topological analysis. Various topological parameters of the PPI network, including the number of edges and nodes, clustering coefficient, network density, degree of freedom, average number of neighbours, network components, characteristic path length and network heterogeneity, were analysed.

### 2.3 Gene Ontology (GO) and Pathway Enrichment Analysis

The gene ontology and pathway enrichment analysis of the common targets with a degree of freedom more than the average of all common targets were analysed using FunRich v3.1.3 software (http://www.funrich.org/). Top ten molecular functions (MF), BPA, cellular components (CC) and Biological processes (BPR) of the screened common targets were identified.

### 2.4 Drug‐Target Molecular Docking

We utilised Maestro software [30] to evaluate the interactions between the top target identified through topological analysis and the 12 selected bioactive compounds. The 3D structures of the ligands were retrieved from the PubChem database (https://pubchem.ncbi.nlm.nih.gov/) and imported into Maestro. Energy minimization was performed on the ligand structures, creating a molecular database in .sdf format. The protein 3D structure (PDB ID: 1I7I) [31] was obtained from the Research Collaboratory for Structural Bioinformatics (RCSB) database (https://www.rcsb.org/) and prepared using the protein preparation wizard in Maestro. Finally, the compounds were docked into the active site using the Dock protocol, and the compound‐target interactions were analysed.

### 2.5 Molecular Dynamic

Molecular dynamics simulations were executed to gain deeper insights into the stability of the most promising molecule under biological conditions. Molecular dynamics studies provide valuable perspectives on complex stability within a solvent system. The system was configured in an orthorhombic box measuring 12 Å × 12 Å × 12 Å, determined using the buffer size approach to minimise box volume. The Desmond system builder implemented a TIP3P solvent model, and the latest OPLS3e force field by Schrodinger Inc. was utilised for modelling proteins and ions [32, 33]. Sodium chloride, with Na + as the cation and Cl‐ as the anion, was incorporated at a concentration of 0.15 M. The Desmond Molecular Dynamics module operated the system for 100 ns, generating approximately 1000 frames. The simulation, conducted in the NPT ensemble class at a stable temperature of 300 K and a pressure of 1 bar, commenced after the system reached a relaxed state [34].

## 3 Results

### 3.1 Common Compound and Disease Target Identification

**FIGURE 1:**
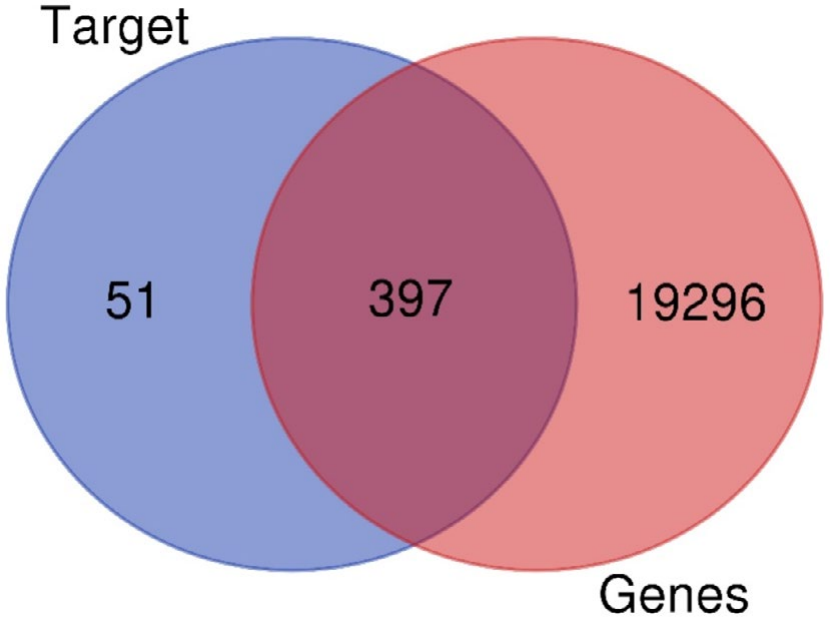
GeneVenn diagram to identify common Disease‐compound targets.

After removing duplicates, we identified 19,693 diabetes‐associated protein targets from the DisGeNET database. We then utilised SwissTargetPrediction to predict potential targets of the ***Gymnema Sylvestre*** compounds, identifying 2306 unique protein targets (Table S1). Following duplicate removal, this was reduced to 448 compound‐associated targets. To find common targets between the disease and compound target datasets, we conducted a GeneVenn diagram analysis (refer to Figure 1), revealing 397 protein targets (see Table S2) jointly targeted by *Bacopa Monnieri* bioactive and implicated in diabetes.

### 3.2 Construction and Analysis of the PPI Network

**FIGURE 2:**
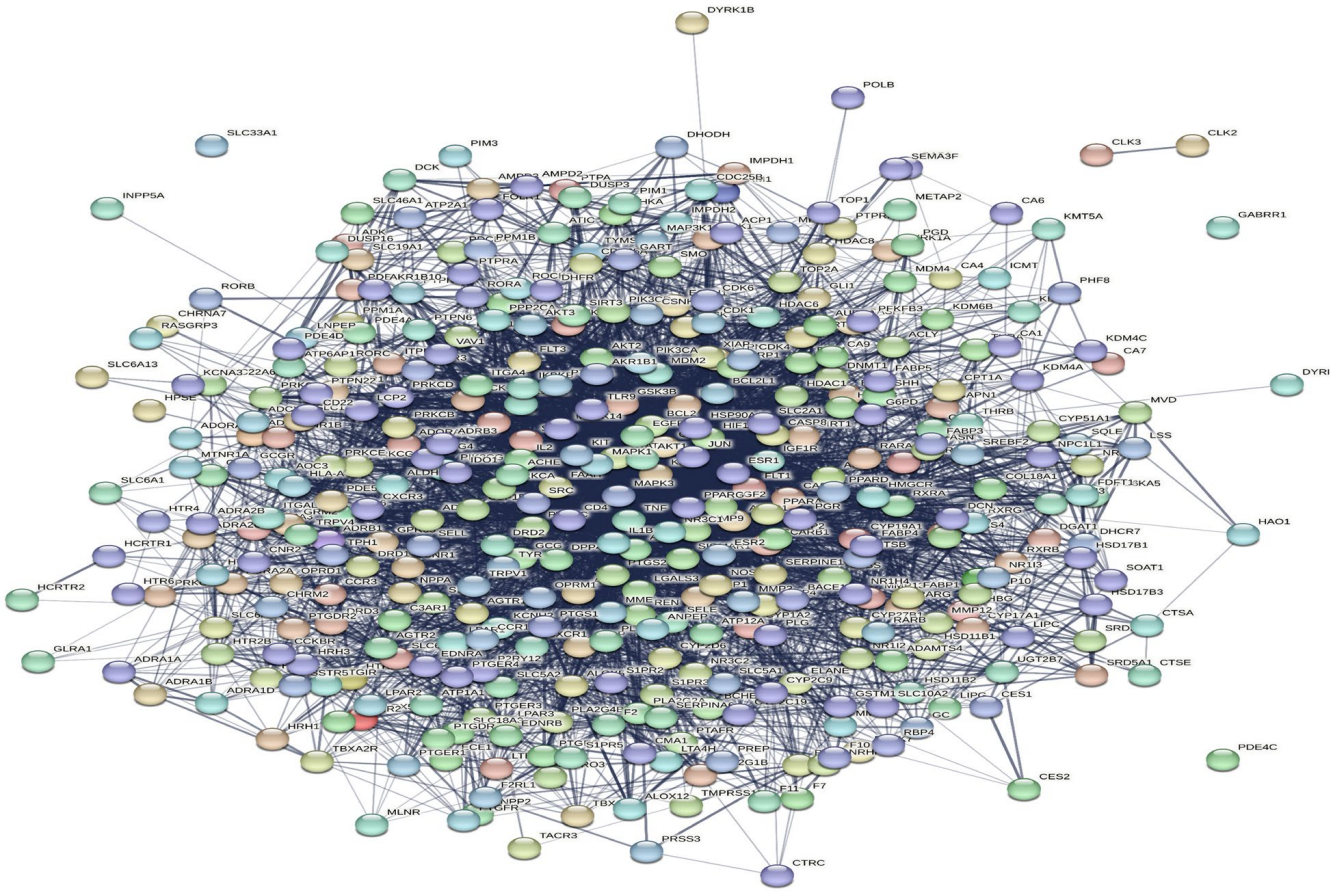
Protein–protein interaction network of screened common targets constructed using STRING database.

The protein–protein interaction network among the common targets was constructed using STRING, as shown in Figure 2.

### 3.3 Analysis of PPI Network

**FIGURE 3:**
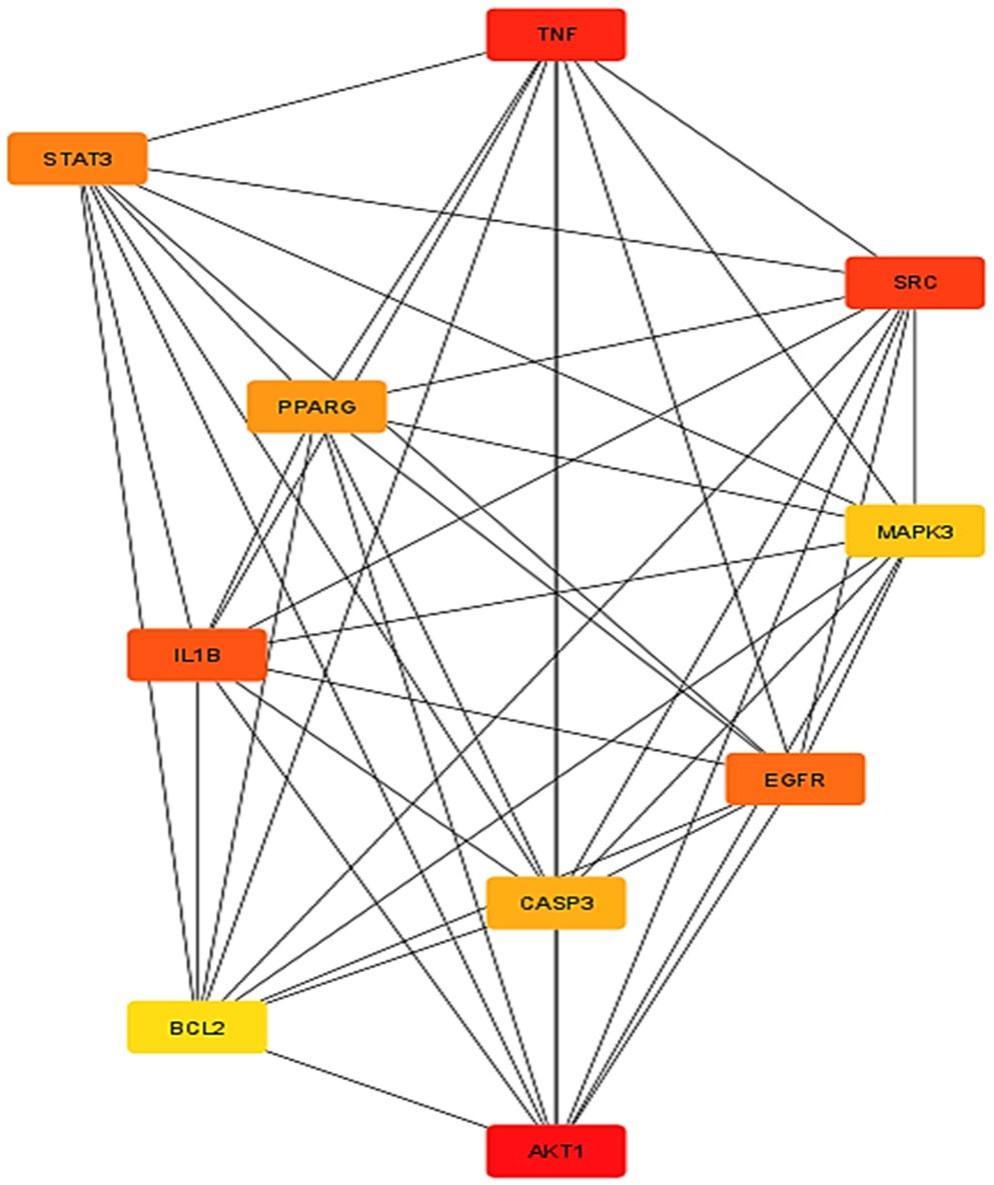
Determine the top 10 genes using topological analysis of protein–protein interactions.

The network analysis was conducted using Cytoscape software, yielding insightful metrics for network characterisation. The resulting network comprised 2601 edges and 401 nodes, with a clustering coefficient of 0.467, network density of 0.03, average number of neighbours at 49.07, network centralization of 0.31, characteristic path length of 2.47 and network heterogeneity of 1.24. In addition, the degree of freedom for each common target is provided in the Table S3. Notably, the average degree was calculated at 49.07, with AKT1 exhibiting the highest degree. Targets surpassing the average degree were selectively employed for subsequent GO and pathway enrichment analyses. The top target, PPARG, identified through topological analysis, was singled out for molecular docking analysis with the selected compounds. Following topological analysis, the top 5 targets were AKT1, SRC, TNF, PPARG and IL1B, indicating their significance in the network and potential functional roles (Figure 3).

### 3.4 Analysing GO and Pathway Enrichment, Along With Conducting KEGG Pathway Analysis

**FIGURE 4:**
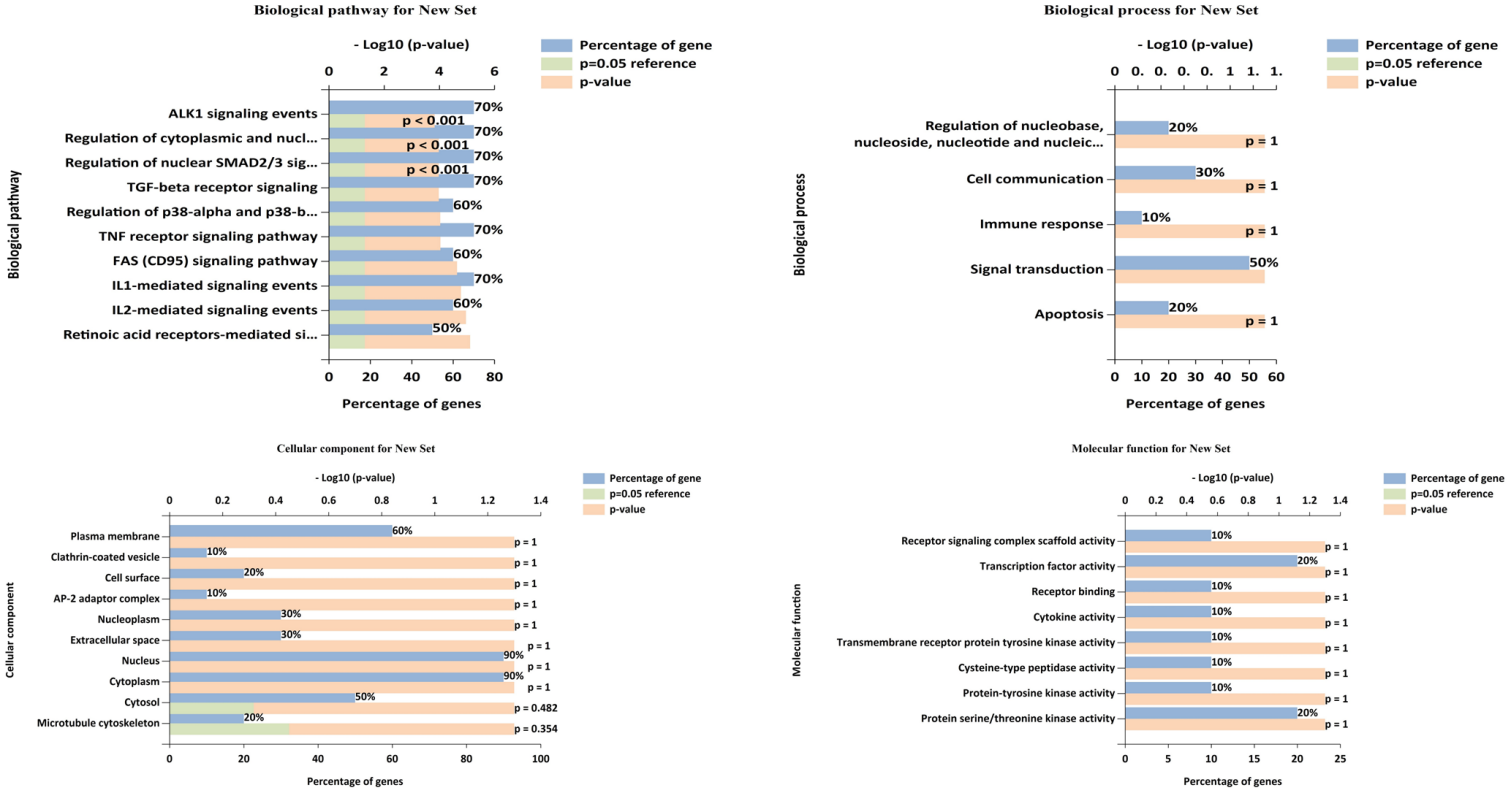
Gene ontology analysis using FunRich software, (a) Cellular components; (b) Molecular function; (c) Biological processes; and (d) Biological pathways.

The analysis of the screened targets has revealed several critical BPA implicated in the progression of the disease. Figure 4a provides an encompassing view of these pathways, emphasising potential targets for preventive interventions. Notably, AKT1 signalling events, associated with cell survival and proliferation, account for approximately 70% of the identified pathways, suggesting a high significance for therapeutic intervention to control abnormal cell growth and enhance apoptosis. The TGF‐beta receptor signalling pathway, constituting a substantial 60%, is known for its regulatory role in cell differentiation and immune response, presenting opportunities to modulate inflammation and potentially impede disease progression. Furthermore, the regulation of nuclear SMAD2/3 signalling, integral to the TGF‐β pathway, becomes a focal point for controlling gene expression and cellular responses, particularly in fibrosis and carcinogenesis. Signalling mechanisms mediated by IL‐1 and IL‐2 play a significant role in immunomodulation by affecting inflammatory processes and activating immune cells. The TNF‐beta receptor and TNF receptor signalling pathways, which account for approximately 70% of inflammation and immunological responses, are prospective targets for regulating cell survival and inflammatory processes. The regulation of cytoplasmic and nuclear activities, which comprise around 70% of recognised pathways, involves several cellular functions affecting cell balance and gene expression. Targeting p‐38‐alpha and p‐38 beta in the MAPK signalling pathways comprising 60% could impact stress and inflammation‐related cellular responses. The FAS (CD95) signalling system is a significant target for initiating apoptosis in abnormal cells. Lastly, the retinoic acid receptors mediated the signalling pathway, influencing cell differentiation and proliferation, accounting for 50%, holding promise for influencing disease progression. Figure 4b illustrates the distribution of target proteins across different BPR, shedding light on their significance. Signal transduction emerges as a predominant biological process, with approximately 50% of the genes associated with it. This underscores the importance of understanding and potentially modulating signalling pathways in the context of disease prevention and progression. Cell communication accounts for a substantial 30%, emphasising the importance of intercellular signalling in disease‐related processes. Apoptosis and the regulation of nucleoside, nucleotide and nucleic acid processes account for almost 20% of the known BPR. The statistical significance is indicated by a *p*‐value of 1, highlighting the strength of these relationships. This indicates a possible function of these systems in influencing disease, especially cell survival and genetic control.

The study of CC revealed informative localization patterns for the selected targets, providing a thorough understanding of their subcellular distribution. As shown in Figure 4c, almost 90% of the tested targets had considerable localization in both the nucleus and the cytoplasm. This dual localization highlights the significance of these proteins in essential physiological functions, such as gene control and intracellular signalling. Furthermore, roughly 60% of the targets are found in the plasma membrane, indicating a potential role in cell‐surface contacts and extracellular signalling. These targets are also localised in the cytosol, which makes up 50% of the cell, indicating that they are involved in various intracellular functions. Extracellular space and nucleoplasm comprise 30% of the targets, suggesting possible involvement in intercellular communication and nuclear activities. Moreover, 20% of the targets are linked to the cell surface, indicating their presence in areas vital for cell‐to‐cell interactions. Clathrin‐coated vesicles, accounting for about 10%, may suggest participation in intracellular transport processes. The microtubule cytoskeleton is localised in 20% of cases with a *p*‐value of 0.354, indicating a possible connection to cellular structural dynamics.

The top 10 functions linked to the screened targets have been identified by studying MF, offering important new understandings of their molecular activities. Figure 4d shows two main roles, each accounting for 20% of the discovered MF: transcription factor activity and protein serine/threonine kinase activity. The functions play a vital role in gene regulation and cellular signalling cascades, indicating the importance of these targets in influencing basic physiological processes. Various functions, each making up 10%, include cytokine activity, receptor binding, receptor signalling complex scaffolds activity, protein‐tyrosine kinase activity, cysteine‐type peptidase activity and transmembrane receptor protein kinase activity.

**FIGURE 5:**
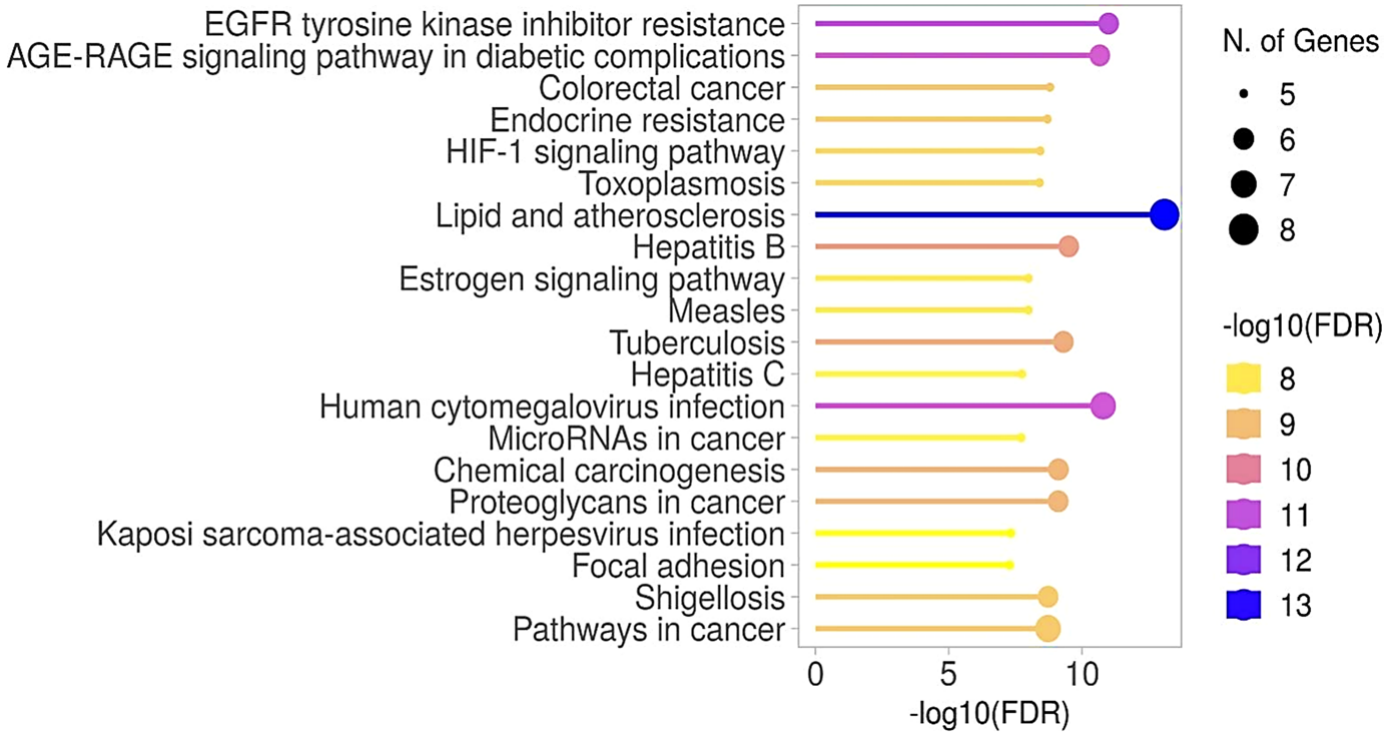
The analysis of KEGG pathways and the genes they interact with.

**FIGURE 6:**
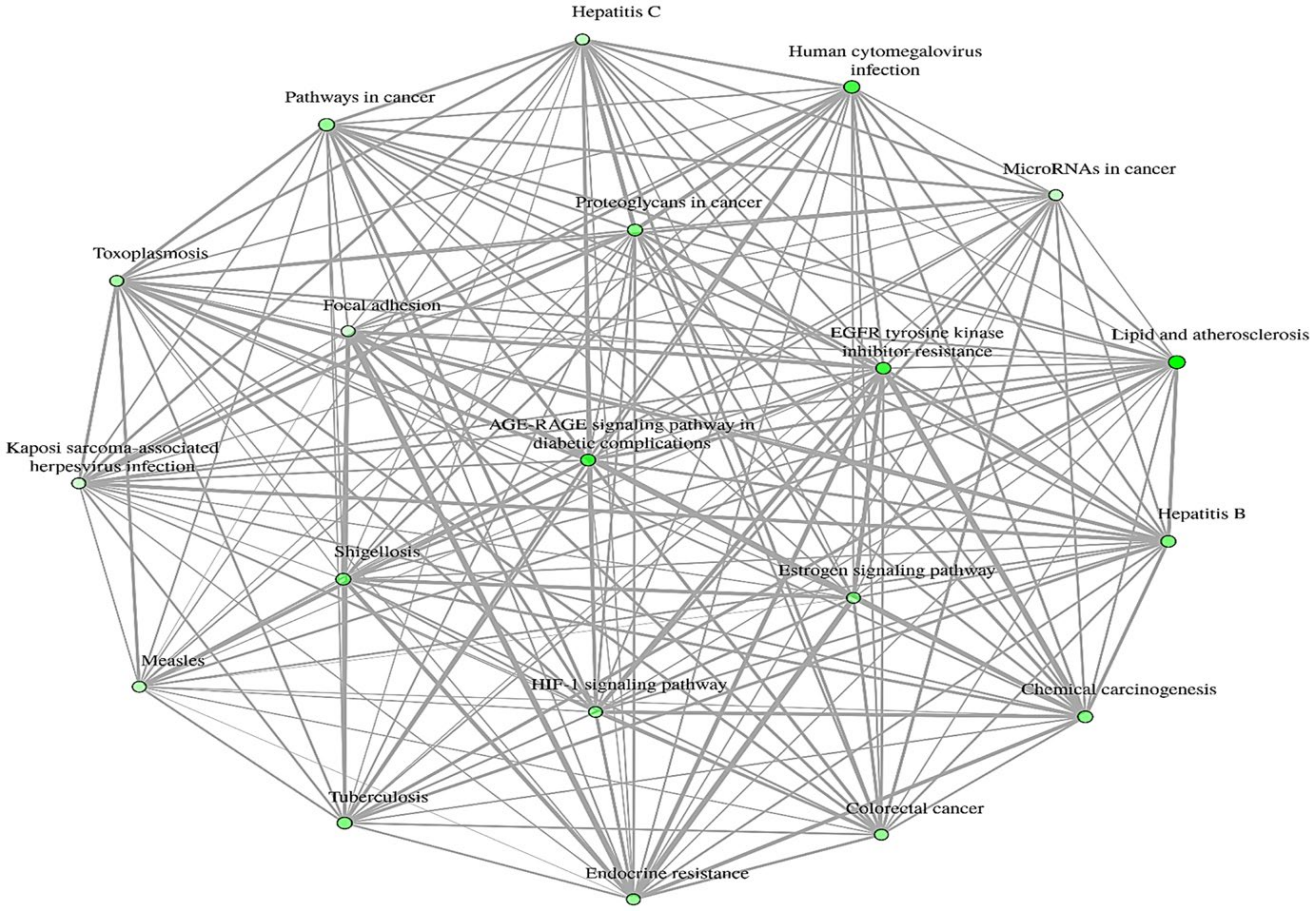
Pathway Enrichment Network Diagram.

The KEGG pathway analysis provides valuable insights into the molecular connections underlying BPR. The Lipid and Atherosclerosis pathway is notable for its many associated genes, indicated by a −log10(FDR) score of around 13. This suggests a strong and statistically significant relationship between the genes in this pathway and the related biological environment. Similarly, the pathway related to EGFR tyrosine kinase inhibitors resistance exhibits a noteworthy −log10 (FDR) value of around 11, emphasising its substantial role and confidence in contributing to resistance mechanisms. The AGE‐RAGE signalling pathway in diabetes complications is also highlighted, with a −log10 (FDR) value of approximately 10, underscoring the strong association between the genes in this pathway and the development of diabetes‐related complications. Additionally, pathways such as Oestrogen Signalling, MicroRNA in Cancer, Focal Adhesion and Kaposi Sarcoma‐Associated Herpesvirus Infection, while showing slightly lower −log10(FDR) values around 8, still exhibit a moderate level of confidence in their respective gene associations (Figures 5 and 6).

### 3.5 Molecular Docking Studies

**FIGURE 7:**
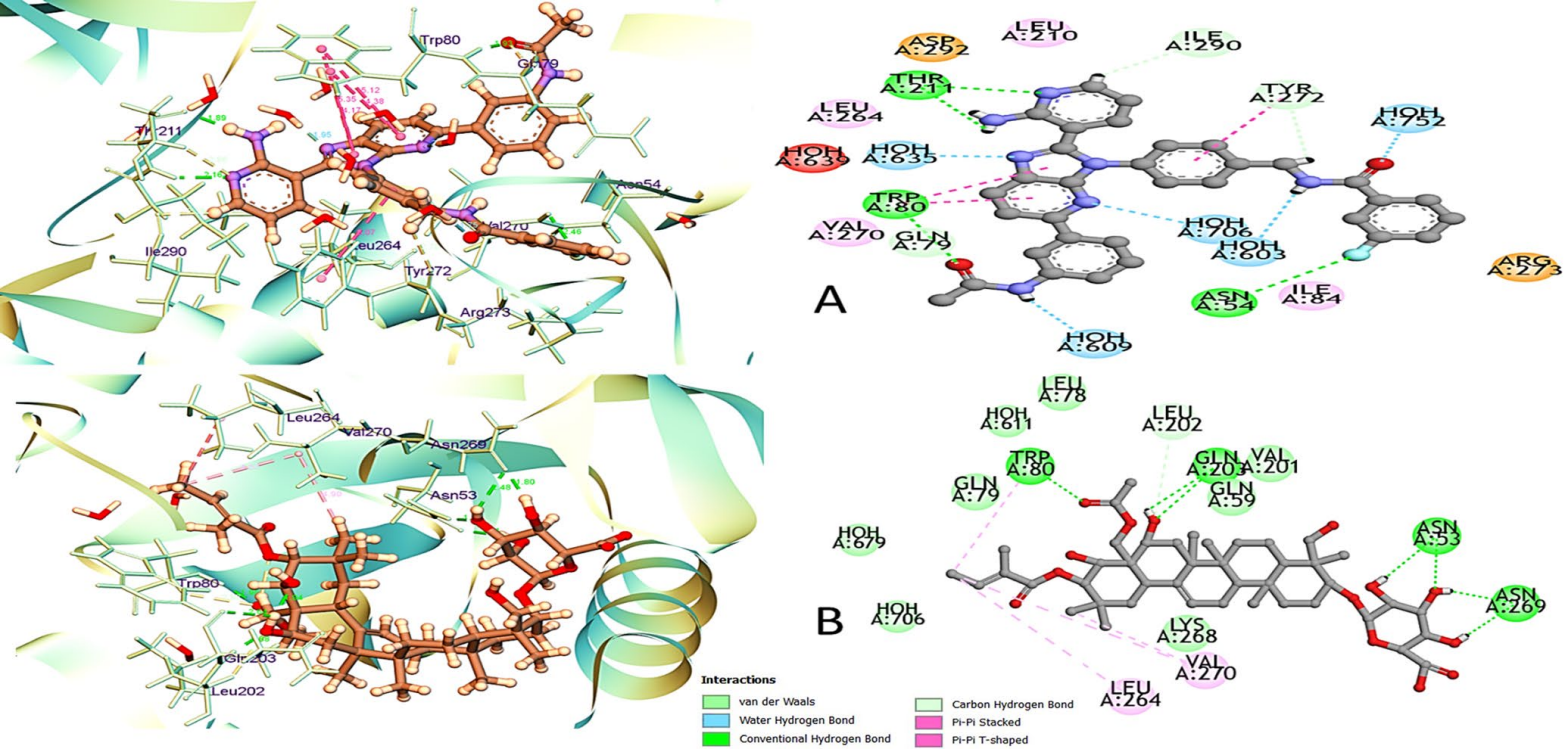
The LigPlot diagram illustrates the protein and ligand complex, with the left side depicting three‐dimensional interactions and the right side illustrating two‐dimensional interactions between the Co‐crystal ligand (A) and Gymnemic acid I (B) to AKT1 interaction.

**FIGURE 8:**
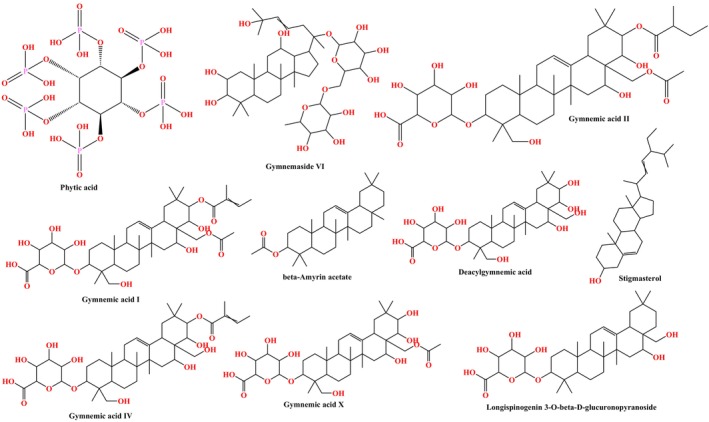
Structure of hit *Gymnema Sylvestre* containing bioactive derivatives.

The top protein identified after topological analysis, i.e., AKT1, was utilised to evaluate the molecular docking studies with the selected ten bioactive constituents of *Gymnema Sylvestre* (Table 1 and Figure 8). The binding energy of compounds with AKT1 in the form of dock score, MMGBSA score and hydrogen and non‐hydrogen bonding interactions is represented in Table 1. *Gymnemic acid I* exhibited a remarkably low binding energy of −9.813 with AKT1, closely approaching the binding energy of the co‐crystal ligand (−9.907). Although the free binding energy (∆G) was marginally higher than that of the co‐crystal ligand, the interaction profile of *Gymnemic acid I* revealed noteworthy features. Notably, *Gymnemic acid I* formed seven hydrogen bond interactions, including Asn53's engagement with the third and fourth positions of the oxygen atom and Asn269's interaction with the 4th and fifth positions of the oxygen atom in the 2H‐pyran ring (Figure 7). Furthermore, the second position of the oxygen atom of icosahydropicene established two hydrogen bonds involving Gln203, and the oxygen atom of the acetate group formed a bond with Trp80. Beyond hydrogen bonds, *Gymnemic acid I* demonstrated pi‐alkyl interactions with Val270, Leu264 and Trp80, particularly with the but‐2‐ene side chain. Additionally, van der Waals interactions were observed around the *Gymnemic acid I* structure, engaging Val201, Leu202, Gln59, Gln79, Leu78 and Lys268. As illustrated in Figure 7, these detailed molecular interactions contribute significant structural insights (Figure 7). These non‐covalent interactions are crucial for fine‐tuning the spatial arrangement and orientation of *Gymnemic acid I* within the binding site, influencing the overall structural stability and, consequently, the binding affinity with AKT1. *Stigmasterol* exhibited a binding affinity of −5.571 and a free binding energy of −30.55 Kcal/mol, as depicted in Table 1. This interaction was primarily attributed to hydrogen bonding with Val201 (see Table S4), along with non‐hydrogen bond interactions involving Gln59, Leu78, Gln79, Trp80, Thr82, Asp292, Leu210, Thr211, Leu264, Lys268, Val270, Val271, Thr272, Asn53‐54, Gln203 and Ser205. On the other hand, *Gymnemaside VI* displayed hydrogen bond interactions with Trp80, water, Asn54 and non‐hydrogen bond interactions with Val201, Leu202, Gln203, Leu78, Gln79, Thr82, Gln59, His194, Leu261, Leu210, Thr211, Leu264, Ile290, Thr291, Asp292, Val270‐71, Tyr272, Kys268 and Asn53. The binding energy associated with *Gymnemaside VI* was −8.079, although it exhibited a lower delta G binding energy across the entire series, measuring −3.03 Kcal/mol (refer to Table 1 and Table S4).

**TABLE 1:** Molecular docking results of *Gymnema Sylvestre* bioactive constituents with AKT1.

Compound Name	Dock score	MMGBSA (∆G) (Kcal/mol)	H‐bond interactions
Co‐Crystal Ligand	−9.907	−60.93	Trp80, Asn54, Thr211
Gymnemic acid I	−9.813	−52.76	Trp80, Gln203, Asn53, Asn269
Stigmasterol	−5.571	−30.55	Val201
Deacylgymnemic acid	−5.935	−26.07	Gln203, water
Beta‐Amyrin acetate	−3.295	−20.76	Water
Longispinogenin	−4.268	−17.72	Trp80, Val201
Gymnemic acid II	−5.981	−17.18	Gln59, water
Gymnemic acid IV	−4.375	−16.88	Water
Gymnemic acid X	−4.478	−5.05	Trp80, water
Gymnemaside VI	−8.079	−3.03	Trp80, water, Asn54
Phytic acid	−5.737	16.95	Trp80, water, Gln203, Asn53

**FIGURE 9:**
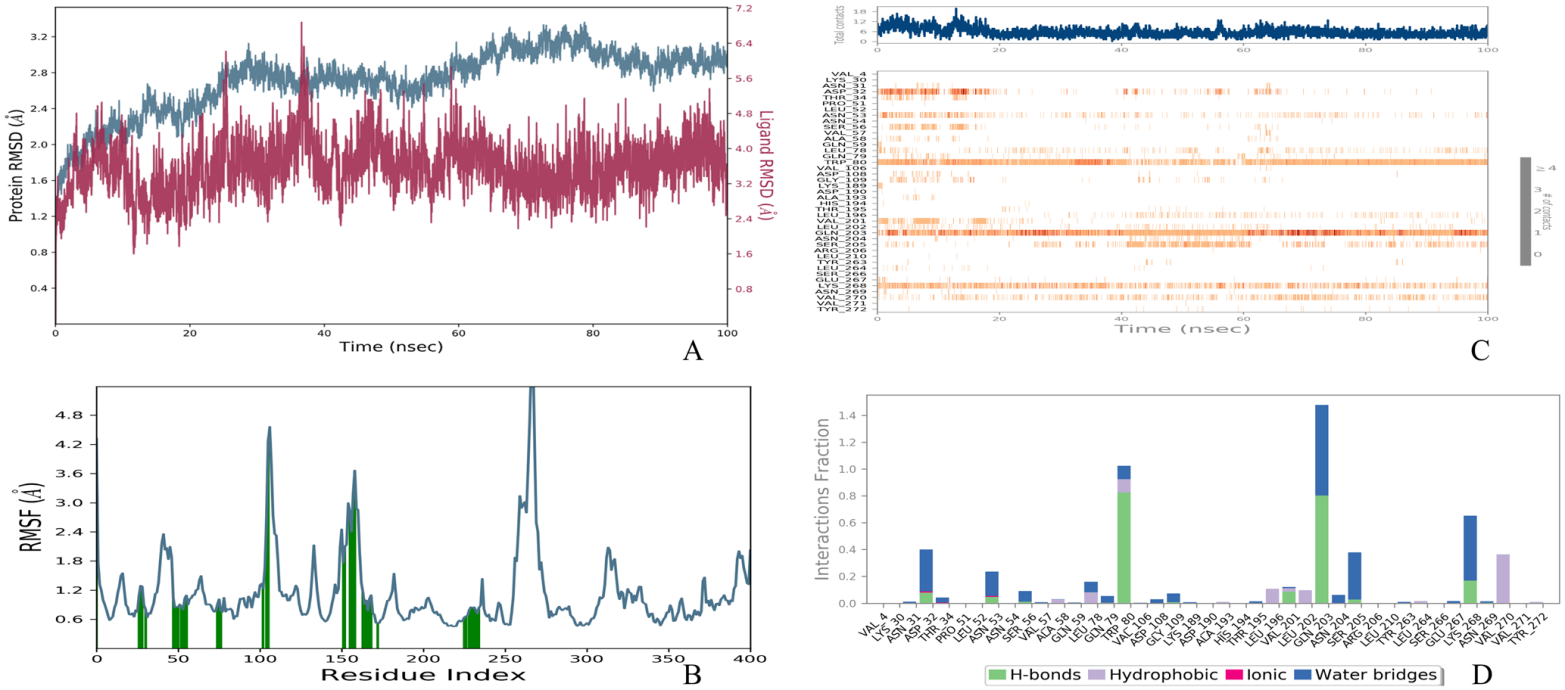
Analysis of Gymnemic Acid I Binding with Target Protein via Molecular Dynamics. (A) Monitoring of structural integrity over time through RMSD, highlighting protein (in blue) and ligand (in red) stability. (B) RMSF analysis reveals residue‐level fluctuations within the complex. (C) Heatmap depiction of consistent protein‐ligand contact points during the simulation. (D) Categorization of interaction types water bridges (blue), hydrogen bonds (green), hydrophobic interactions (purple) and ionic bonds (pink)—in a comprehensive histogram.

### 3.6 Molecular Dynamic

The binding interaction between a protein and the ligand Gymnemic acid I was examined through MD simulations. Gymnemic acid I reveals remarkable stability, with consistent Root Mean Square Deviation (RMSD) values for C‐alpha atoms maintaining 2.4 Angstroms from 65 to 100 ns. Conformational shifts occurred between 30 and 40 ns, but the ligand returned to a stable state after that. Root mean square fluctuation (RMSF) values revealed substantial fluctuations in the protein's loop regions and termini, while lower RMSF values were seen at the binding site, indicating a robust interaction with Gymnemic acid I. Structural analysis showed alpha‐helices and beta‐strands collectively contributed 41.62% to the protein's structure, with helices and strands comprising 20.09% and 21.53%, respectively. This mix of secondary structures likely contributes to the protein's overall stability. The robustness of the interaction between the protein and drug candidates was further examined by quantifying the number and length of hydrogen bonds throughout the simulation (Figure 9). The hydrogen bond strengths were also categorised by measuring the distance between the hydrogen bond donor and acceptor atoms. Hydrogen bonds with a donor‐acceptor distance of 2.2–2.5 Angstroms were classified as strong bonds, distances of 2.6–3.2 Angstroms were considered moderate strength, and those with 3.2–4 Angstroms were categorised as weak hydrogen bond interactions. Key hydrogen bonding interactions were identified between the ligand and residues Trp80 (with the 3rd OH group at 80% occupancy), Gln203 (with the second oxygen atom of icosahydropicene at 43% occupancy), and Lys268, at an average distance of 2.5 Angstroms. The solvent‐accessible surface area (SASA) provides a quantitative assessment of the surface of a molecule that can come into contact with solvent molecules. A relatively large SASA value reflects a molecular structure where a significant portion of the surface is exposed to potential solvent interactions. Solvent‐accessible surface area and radius of gyration (RG) profiles showed that after a 65 ns equilibration period, the Gymnemic acid‐protein complex remained stable in structure and size over the remaining 100 ns simulation (see Table S5). The RG assesses a molecule's or molecular complex's overall size. Higher RG values correlate with larger, more expansive molecular structures. In contrast, lower RG values are associated with smaller, more compact molecule structures. Tracking RG over time can reveal changes in the size and compactness of a molecule's structure caused by dynamic activities such as folding or binding. The RG profiles for Gymnemic acid I complexes show a consistent degree of 6.4 Angstroms throughout the simulation period (see Table S5). These data imply that Gymnemic acid I complexes are stable, maintaining their structure and size over a lengthy simulation period, thereby verifying their potential for sustained binding to protein complexes.

## 4 Discussion

The increasing global incidence of diabetes and its related health issues highlights the urgent need for new treatment approaches. *Gymnema Sylvestre* , a herbal plant known for its powerful antioxidant, anti‐inflammatory and glucose‐lowering effects, presents a significant opportunity to explore its therapeutic properties against diabetes (2–3). In this research, we utilised computational methods to examine the complex interactions between the active compounds of *Gymnema Sylvestre* and diabetic pathways. We focused on ten active compounds such as Gymnemic acid I, Stigmasterol, Deacylgymnemic acid, Beta‐Amyrin acetate, Longispinogenin, Gymnemic acid II, Gymnemic acid, Gymnemaside VI, Phytic acid and Gymnemic acid X. Our study identified 397 common targets linking these compounds with diabetes, laying the groundwork for in‐depth investigation. Through GO analysis, we investigated the associated CC, MF, BPR and pathways of these targets, providing insights into the potential anti‐diabetic mechanisms of *Gymnema Sylvestre* . Integrating GO parameters has significantly deepened our comprehension of the biological activities, molecular functionalities and cellular locations affected by the active components of *Gymnema Sylvestre* , especially in their engagement with the protein target AKT1. The molecular functionalities influenced by these components shed light on the conceivable pathways through which they exert anti‐diabetic effects. Prominent molecular functionalities, including ‘enzyme binding’ and ‘receptor binding,’ highlight the precise nature of interactions with AKT1 and similarly important proteins. This specificity provides crucial information on how these compounds selectively target molecular pathways, offering insights into their potential therapeutic actions against diabetes.

Delving into the CC influenced by the constituents of *Gymnema Sylvestre* is essential for understanding their intracellular distribution and potential effects on cellular architecture. The comprehensive analysis of the pinpointed gene targets illuminated numerous pivotal pathways and processes involved in disease progression, highlighting areas that might benefit from therapeutic exploration. Among the notable discoveries, the modulation of the AKT1 and TGF‐beta signalling pathways appears crucial, affecting cell survival, proliferation, differentiation and fibrosis. Addressing the TNF receptor pathways and immune signalling mechanisms could also offer ways to manage inflammation effectively. Within the realm of AKT1 signalling, genes linked with uncontrolled cell growth and increased apoptosis stand out, underlining their significance as potential therapeutic targets. In the context of TGF‐beta receptor signalling, genes that govern cell differentiation and immune responses present precise intervention opportunities to control inflammation and slow disease advancement. The central roles of AKT1 signalling and TGF‐beta receptor signalling, contributing 70% and 60% to the identified targets, respectively, underscore their importance. AKT1 signalling is crucial for cell survival and proliferation, whereas TGF‐beta receptor signalling focuses on cell differentiation and immune modulation.

Furthermore, due to their extensive gene contributions, the TNF‐beta receptor and IL‐1/IL‐2‐mediated signalling pathways shed light on possible targets for adjusting inflammatory responses and activating immune cells. The involvement of specific genes in the p‐38‐alpha and p‐38‐beta within the MAPK signalling pathways indicates their importance in mediating stress and inflammation responses. The analysis of CC reveals the gene distribution across different subcellular locations, showcasing their roles in key cellular functions. Genes related to transcription factor activity and protein serine/threonine kinase activity are crucial at the molecular level, affecting gene expression and signalling pathways. KEGG pathway analysis further clarifies the genes' involvement in particular pathways, with significant links to the Lipid and Atherosclerosis pathway, resistance against EGFR tyrosine kinase inhibitors, and the AGE‐RAGE signalling pathway in diabetic complications, highlighting their potential impact on disease progression and therapeutic strategies.

The serine/threonine kinase Akt, also known as protein kinase B (PKB), is a key regulator of glucose and lipid energy metabolism, making it a critical subject of study in diabetes and metabolic disorders. It is primarily found in organs essential to metabolism and becomes activated through various triggers, including cellular stress, physical activity and various hormones and pharmaceuticals that affect cellular metabolic processes. This enzyme's activation is a significant contributor to managing the body's metabolic pathways, highlighting its importance in both normal physiological conditions and the pathogenesis of metabolic diseases. Genetic and pharmacological investigations have underscored the indispensability of Akt in maintaining the equilibrium of glucose and lipid metabolism and overseeing a spectrum of cellular responses. Compelling evidence establishes a correlation between metabolic syndrome and insulin resistance and lipid metabolism disorders. Drawing from an extensive body of research on Akt‐related pathways and reactions, it is plausible to consider Akt as a promising drug target for the effective treatment of metabolic syndrome [35, 36]. Integrating GO parameters with molecular docking results provides a comprehensive view of how the bioactive constituents exert their effects at the molecular and cellular levels. For instance, the enrichment of terms related to the ‘glycolytic process’ corresponds well with the observed interactions of Gymnemic acid I, suggesting a potential disruption of glycolytic pathways through its binding to AKT1.

Interpretation of the molecular docking results reveals that lower dock scores and greater hydrogen bonds correspond to stronger binding between the ligand and protein. The favourable binding energy of −9.813 kcal/mol for Gymnemic Acid I indicates robust affinity for AKT1, suggesting it may play a role in modulating glycolytic pathways linked to diabetes. Specific hydrogen bonding with residue Asn269 and additional non‐covalent interactions with multiple residues highlight the selective nature of Gymnemic Acid I binding to AKT1. This supports the potential for Gymnemic Acid I to disrupt glycolytic processes connected to diabetes progression. Studies have shown that Gymnemic Acid I protects MIN‐6 cells from high glucose‐induced apoptosis through mTOR inhibition [37]. Molecular dynamics simulations provided comparative insights into the distinct behaviours of Gymnemic Acid I versus a co‐crystal ligand when bound to the protein. Gymnemic Acid I demonstrated consistent stability per RMSD measurements, a unique equilibration phase, and defined conformational shifts. The balanced alpha‐helix and beta‐strand composition and key hydrogen bonds involving Trp80, Gln203 and Lys268 residues contribute to stability. By applying network pharmacology and MD simulations, this study contributes preliminary insights into the interaction dynamics of *Gymnema Sylvestre* compounds with diabetes‐related targets. While previous studies typically focus on individual methodologies, this dual approach offers observations on both target identification and compound stability within biological systems, which may support further research in understanding these interactions.

### 4.1 Limitations and Prospects for Future Exploration

While GO analysis offers valuable insights into the BPR, MF and CC associated with *Gymnema Sylvestre's* bioactive constituents, it's crucial to acknowledge the method's inherent limitations. A significant constraint is the potential bias in existing annotations, which might not fully encompass the entire range of cellular activities. These annotations are contingent on the prevailing scientific knowledge, potentially omitting aspects of cellular functions and leading to uncertainties in interpreting GO analysis outcomes. Moreover, cellular processes' dynamic and complex nature, which can vary over time or in response to particular stimuli, presents additional challenges. Such complexities may not be fully captured by static GO annotations, complicating the prediction of outcomes based solely on bioinformatics analysis. Future research should integrate experimental validation methods to overcome these limitations and bolster the credibility of the findings. Conducting in vitro studies allows for meticulously investigating cellular reactions to *Gymnema Sylvestre's* constituents under controlled conditions. These studies can provide empirical support or adjustments to the predicted GO terms, enhancing our comprehension of these components' effects on cellular mechanisms implicated in diabetes.

Furthermore, in vivo studies performed in living organisms are essential for corroborating and contextualising the effects observed in bioinformatics analyses. Therefore, as part of our future work, we plan to conduct additional wet lab experiments to validate our computational predictions. Specifically, we intend to perform in vitro studies to examine cellular responses, followed by in vivo studies in suitable animal models to assess pharmacokinetics, efficacy and safety. Such studies will enable us to evaluate how *Gymnema Sylvestre* compounds interact within complex biological systems, providing a more comprehensive perspective on their therapeutic effects and limitations in managing diabetes. This approach will bridge the gap between computational insights and practical application, facilitating a more informed understanding of *Gymnema Sylvestre's* role in therapeutic interventions.

## 5 Conclusion

In conclusion, our study explores the potential of *Gymnema Sylvestre* derivatives in managing diabetes, particularly through their involvement in various disease pathways, which may support drug development efforts. By employing network pharmacology and in silico modelling, we identified cellular targets such as AKT1, TNF, SRC, IL1B and EGFR, which are associated with cell survival, inflammation and proliferation. Gymnemic acid I was observed to have a binding energy of −9.813 with AKT1, approaching the binding affinity of the co‐crystal ligand (−9.907), and exhibited stability in MD simulations, mainly because of hydrogen bonding with residues Trp80, Gln203 and Lys268. These findings provide a basis for the compound's stability and highlight the intricate interactions that contribute to its therapeutic potential. These preliminary findings indicate interactions that could be relevant for therapeutic applications; however, further in vitro and in vivo studies are necessary to understand better the efficacy and safety of *Gymnema Sylvestre* derivatives in diabetes management.

## Author Contributions

**Amal Mayyas:** methodology (equal), writing – original draft (equal). **Ali Al‐Samydai:** formal analysis (equal), writing – original draft (equal). **Amjad Ibrahim Oraibi:** data curation (equal), writing – original draft (equal). **Nawres Debbabi:** visualization (equal), writing – original draft (equal). **Sara S. Hassan:** software (equal), writing – original draft (equal). **Hany Aqeel Al‐Hussainy:** resources (equal), writing – original draft (equal). **Ahmad Mohammad Salamatullah:** supervision (equal), writing – original draft (equal). **Musaab Dauelbait:** conceptualization (equal), writing – original draft (equal). **Mohammed Bourhia:** writing – original draft (equal), writing – review and editing (equal). **Khalid S. Almaary:** formal analysis, reviewing and Editing.

## Consent

The authors have nothing to report.

## Conflicts of Interest

The authors declare no conflicts of interest.

## Supporting information


Appendix S1.

## Data Availability

All data generated or analysed in this study are included within the manuscript and its supplementary files. Additional data may be available upon request from the corresponding author.
