# Supplementary material for: Deciphering the Anti‐Diabetic Potential of Gymnema Sylvestre Using Integrated Computer‐Aided Drug Design and Network Pharmacology
Source: J Cell Mol Med. 2025 Jan 14;29(1):e70349. doi: 10.1111/jcmm.70349 (PMC11733079; doi:10.1111/jcmm.70349)
Supplement: Supplementary file 1 — Appendix S1. [file JCMM-29-e70349-s001.docx]

**Supplementary file I**

**Deciphering the Antidiabetic Potential of Gymnema Sylvestre Using Integrated Computer aided drug design and network pharmacology**

**Table s1:** SwissTargetPrediction to predict potential targets of the Gymnema Sylvestre compounds (2,306 unique protein targets).

| F2RL1 | FABP3 | PRKCQ | ADCY1 | S1PR4 | BCL2 | F2 |
| --- | --- | --- | --- | --- | --- | --- |
| ITGA2B ITGB3 | FABP5 | HSD11B2 | FOLH1 | PLA2G4A | SELL | GLI1 |
| OPRK1 | FABP1 | HSD11B1 | S1PR5 | PLA2G4B | SELE | JUN |
| TTL | PGR | RORC | S1PR4 | TRPV1 | DHODH | PRKCA |
| ITGB5 ITGAV | SLC6A3 | ATP1A1 | S1PR3 | PTPN22 | ADAMTS4 | STAT3 |
| ITGAV ITGB6 | ADORA3 | PTPA | S1PR1 | SLC10A2 | ITGB5 ITGAV | KCNA3 |
| PTPN1 | MAPK3 | PPM1B | AOC3 | PFKFB3 | ITGAV ITGB6 | FDFT1 |
| NOS2 | PRKCH | PPP1CC | SQLE | CASP3 | SQLE | RORC |
| ADRB2 | PTPN11 | PPP2CA | AKT1 | CXCR1 | GRB2 | PRKCD |
| FOLH1 | AKR1B10 | PPP2R5A | HTR2B | NR3C2 | GCG | PRKCB |
| PPM1A | ICMT | SQLE | ADRA2A | SERPINA6 | S1PR5 | PRKCE |
| GART | MDM2 | TERT | ADRA2C | FOLH1 | ATIC | PRKCH |
| IARS | CXCR3 | PTPN1 | ADRA2B | EGLN1 | S1PR4 | PRKCQ |
| TLR9 | S1PR3 | PTGS2 | DRD1 | GCG | S1PR3 | PPM1B |
| STAT3 | S1PR1 | PSEN2 PSENEN NCSTN APH1A PSEN1 APH1B | DRD2 | IL2 | S1PR1 | PTPN1 |
| DHODH | SMO | PRKCA | ADRA1D | AKT2 | BACE1 | PPP1CC |
| PRKCG | HCRTR2 | NOS2 | HTR2A | AKT1 | CYP19A1 | PPP2CA |
| VAV1 | HCRTR1 | PDE4D | HTR2C | AKT3 | TTL | PPP2R5A |
| TRPV4 | PTPRF | F2RL1 | ADRA1A | PTGER1 | TBXAS1 | VDR |
| PCSK7 | PLA2G1B | ATP2A1 | CYP2D6 | AOC3 | FFAR1 | ATP2A1 |
| TYMS | ACP1 | PRKCG | HTR6 | PDCD4 | ATP2A1 | GLRA1 |
| PFKFB3 | NR3C1 | VAV1 | HTR1B | PDE4B | CASP3 | GLRA2 |
| LTB4R | CCR1 | TRPV4 | GART | EDNRA | FPGS | SQLE |
| PTGDR2 | METAP1 | PPM1A | MMP9 | ANPEP | PRSS3 | TERT |
| PRKCA | GC | FOLH1 | ATP2A1 | LNPEP | MMP9 | OPRM1 |
| ADCY1 | TOP2A | SLC2A1 | DHODH | GCK | CTRC | OPRK1 |
| DPP4 | MDM4 | ADCY1 | PDE5A | LTA4H | IARS | AR |
| F10 | PIM1 | PGGT1B FNTA | ITGA2B ITGB3 | DHFR | ITGB1 ITGA5 | NR3C1 |
| GRM2 | PIM3 | GLRA1 | LTA4H | ESR1 | PTGDR2 | MMP9 |
| PTAFR | SHH | GLRA2 | MMP2 | HSD11B2 | PTPN2 | PTGS2 |
| GSTM1 | PTGFR | TTL | GCG | HSD11B1 | HMGCR | PTPA |
| SRC | PTGER3 | ADORA2A | PTPN2 | BCL2L1 | HSD11B2 | F2RL1 |
| PTPN22 | PTGDR | CASP3 | DHFR | F2 | HSD11B1 | PPM1A |
| PLA2G1B | PDE4D | STAT3 | TPH1 | GLI1 | BCL2L1 | PSEN2 PSENEN NCSTN APH1A PSEN1 APH1B |
| TRPV1 | TOP1 | TYMS | ITGA2 ITGB3 | JUN | F2 | TTL |
| CCKBR | NR3C2 | LTB4R | PFKFB3 | PRKCA | GLI1 | PDE4D |
| CXCR1 | HIF1A | NR3C1 | BACE1 | STAT3 | JUN | IARS |
| PRSS3 | CDK6 | S1PR5 | PTGER1 | KCNA3 | PRKCA | PTAFR |
| NR3C2 | CDK4 | PTPN22 | CTRC | FDFT1 | STAT3 | PCSK7 |
| TMPRSS15 | SRD5A2 | S1PR4 | SLC46A1 | RORC | KCNA3 | GSTM1 |
| SERPINA6 | F2 | S1PR3 | FOLR1 | PRKCD | FDFT1 | HMGCR |
| DHFR | ITGAL ICAM1 ITGB2 | S1PR1 | SLC19A1 | PRKCB | RORC | IKBKB |
| PDCD4 | F10 | PTAFR | HSD11B2 | PRKCE | ATP1A1 | SLC2A1 |
| PTPRA | GNRHR | FFAR1 | HSD11B1 | PRKCH | PRKCD | ADCY1 |
| SLC46A1 | DGAT1 | DHFR | F2 | PRKCQ | PRKCB | TLR9 |
| FOLR1 | HSD11B1 | TLR9 | GLI1 | PPM1B | PRKCE | FNTA FNTB |
| PTPN6 | BCL2L1 | SSTR5 | JUN | PTPN1 | PRKCH | PGGT1B FNTA |
| FFAR1 | HSD11B2 | ITGB1 ITGA4 | FDFT1 | PPP1CC | PRKCQ | FGF1 |
| ADRA1B | AR | ADORA3 | VDR | PPP2CA | PPM1B | GSK3B |
| CCR6 | PTPN1 | ADRB2 | NR3C1 | PPP2R5A | PTPN1 | ADORA1 |
| SOAT1 | MMP9 | PRKCE | PTPN1 | VDR | PPP1CC | ITPR3 |
| DGAT1 | GLI1 | PCSK7 | PPM1A | ATP2A1 | PPP2CA | ITPR1 |
| MLNR | STAT3 | ATP6AP1 | AR | GLRA1 | PPP2R5A | INPP5A |
| PLG | F2 | TBXAS1 | GLRA1 | GLRA2 | VDR | PGD |
| SLC6A4 | VDR | SLC46A1 | GLRA2 | SQLE | ATP2A1 | CDC25A |
| ATP6AP1 | IL2 | FOLR1 | OPRK1 | TERT | GLRA1 | CDC25B |
| NR3C1 | NR3C1 | ADRB1 | TLR9 | OPRM1 | GLRA2 | LPAR3 |
| CSNK2A1 | PTGER1 | SLC19A1 | HMGCR | OPRK1 | SQLE | LPAR1 |
| ABCB1 | FDFT1 | MLNR | PTPA | AR | TERT | AKT1 |
| ITGAV ITGB1 | GART | TRPV1 | PTAFR | NR3C1 | OPRM1 | FOLH1 |
| SERPINE1 | PDE5A | EGLN1 | BCL2L1 | MMP9 | OPRK1 | GPR55 |
| IL1B | CASP3 | CXCR1 | HSD11B2 | PTGS2 | AR | PDPK1 |
| F7 | CASP1 | ANPEP | HSD11B1 | PTPA | NR3C1 | MTOR |
| TPH1 | RORC | LNPEP | F2 | F2RL1 | MMP9 | LPAR6 |
| GCG | TYMS | HRH1 | GLI1 | PPM1A | PTGS2 | LPAR2 |
| TBXAS1 | ADK | CCR3 | JUN | PSEN2 PSENEN NCSTN APH1A PSEN1 APH1B | PTPA | LPAR5 |
| S1PR2 | PDE4A | SF3B3 | VDR | TTL | F2RL1 | LPAR4 |
| S1PR4 | PDE4B | SCARB1 | FDFT1 | PDE4D | PPM1A | IGF2R |
| S1PR3 | CASP7 | SLC10A2 | PSEN2 PSENEN NCSTN APH1A PSEN1 APH1B | IARS | PSEN2 PSENEN NCSTN APH1A PSEN1 APH1B | ENPP2 |
| S1PR1 | PPM1B | GART | PPM1A | PTAFR | TTL | MVD |
| BCL2 | PPP2R5A | ALDH2 | GLRA1 | PCSK7 | PDE4D | RORC |
| OPRM1 | SYK | F11 | GLRA2 | GSTM1 | IARS | HSD11B2 |
| ITGB1 ITGA4 | CTSA | PLA2G4B | OPRK1 | HMGCR | PTAFR | STAT3 |
| HSD11B2 | PTAFR | GSTM1 | AR | IKBKB | PCSK7 | HTR2B |
| HSD11B1 | ITGA2B ITGB3 | PLA2G1B | NR3C1 | SLC2A1 | GSTM1 | ADRA2B |
| BCL2L1 | CASP6 | GRM2 | MMP9 | ADCY1 | HMGCR | DRD1 |
| JUN | CASP8 | F10 | PTAFR | TLR9 | IKBKB | ADRA1D |
| GLI1 | DHFR | SERPINE1 | PRKCA | FNTA FNTB | SLC2A1 | HTR2C |
| KCNA3 | MMP2 | GCG | FNTA FNTB | PGGT1B FNTA | ADCY1 | CYP2D6 |
| FDFT1 | GRB2 | F3 F7 | TLR9 | NOS2 | TLR9 | HTR1B |
| PRKCD | MMP10 | GRB2 | PTPA | PLA2G1B | FNTA FNTB | ATP1A1 |
| PRKCB | MMP12 | METAP2 | IL2 | TRPV1 | PGGT1B FNTA | PTAFR |
| PRKCE | FFAR1 | ELANE | METAP2 | OPRD1 | NOS2 | IL2 |
| PRKCH | MMP8 | SELL | HMGCR | TRPV4 | PLA2G1B | AR |
| PRKCQ | PPP2CA | SELE | PPP1CC | SF3B3 | TRPV1 | NR3C1 |
| RORC | AMPD2 | HMGCR | PTPN2 | CXCR1 | OPRD1 | VEGFA |
| ATP1A1 | SELL | HLA-A | PPM1B | PRKCG | TRPV4 | FGF1 |
| F2 | SELE | PTGER1 | PPP2CA | VAV1 | SF3B3 | FGF2 |
| PPM1B | HTR2B | CSF1R | PPP2R5A | IL2 | CXCR1 | HPSE |
| PPP2CA | ADRA2A | KIT | OPRM1 | METAP2 | PRKCG | PSEN2 PSENEN NCSTN APH1A PSEN1 APH1B |
| PPP2R5A | ADRA2C | FLT3 | KCNA3 | EDNRA | VAV1 | TYMS |
| PTPA | ADRA2B | HSD11B2 | PRKCD | ATP1A1 | IL2 | BCL2L1 |
| ATP2A1 | DRD1 | HSD11B1 | PRKCB | GCGR | METAP2 | LGALS4 |
| SQLE | DRD2 | BCL2L1 | PRKCE | ADRA1A | EDNRA | LGALS3 |
| TERT | ADRA1D | F2 | PRKCH | ADRA1B | GCGR | LGALS8 |
| AR | HTR2A | JUN | PRKCQ | CCR6 | ADRA1A | TLR9 |
| PTGS2 | HTR2C | GLI1 | PTPN1 | NR3C2 | ADRA1B | VDR |
| GLRA1 | ADRA1A | PRKCA | HTR2B | ABCB1 | CCR6 | PTPA |
| GLRA2 | DRD3 | RORC | ADRA2A | DHFR | NR3C2 | GLRA1 |
| ITGAV ITGB3 | CYP2D6 | ATP1A1 | ADRA2C | SERPINA6 | ABCB1 | GLRA2 |
| RBP4 | HTR6 | FDFT1 | ADRA2B | IL1B | SERPINA6 | PTPN1 |
| ADORA1 | HTR1B | KCNA3 | DRD1 | PTPN2 | IL1B | GLI1 |
| ADORA2A | PLG | PRKCD | DRD2 | PDCD4 | PTPN2 | KDR |
| ADORA3 | PRSS3 | PRKCB | ADRA1D | P2RY10 | PDCD4 | JUN |
| AR | ACE | PRKCE | HTR2A | GPR174 | DHFR | DRD2 |
| CYP19A1 | ITGB1 ITGA4 | PRKCH | HTR2C | MLNR | MLNR | DRD3 |
| ESR1 | JUN | PRKCQ | ADRA1A | PLA2G2A | FPGS | GRB2 |
| ESR2 | AKR1B1 | PPM1B | DRD3 | ATP6AP1 | PLA2G2A | HSD11B1 |
| RORC | F3 F7 | PPP1CC | CYP2D6 | CYP19A1 | ATP6AP1 | ITGAV ITGB3 |
| HTR2B | IMPDH1 | PPP2CA | HTR6 | CNR2 | CYP19A1 | PTGS2 |
| RXRB | MME | PPP2R5A | HTR1B | HTR2B | CNR2 | PPM1A |
| ADRA2B | IMPDH2 | AR | TERT | ADRA2A | TYMS | MMP9 |
| RARG | F10 | PSEN2 PSENEN NCSTN APH1A PSEN1 APH1B | TYMS | ADRA2C | HTR2B | CES2 |
| RXRG | PPM1A | TTL | MLNR | ADRA2B | ADRA2A | HSP90AA1 |
| RARB | ITGB7 ITGA4 | PTPA | PTGS2 | DRD1 | ADRA2C | PRKCD |
| RARA | ITGAV ITGB3 | ATP2A1 | F2RL1 | PRSS1 | ADRA2B | PRKCQ |
| RXRA | ITGB5 ITGAV | GLRA1 | STAT3 | DRD2 | DRD1 | ADRA2A |
| MAPK14 | SERPINE1 | GLRA2 | FGF1 | ADRA1D | PRSS1 | ADRA2C |
| RORB | TBXAS1 | SQLE | SLC2A1 | HTR2A | DRD2 | FDFT1 |
| MAPK1 | HDAC6 | DHFR | ADCY1 | HTR2C | ADRA1D | PRKCB |
| RORA | ALDH2 | KDR | NR3C2 | DRD3 | HTR2A | PRKCE |
| CHKA | ESR1 | PTGS2 | SERPINA6 | CTRC | HTR2C | PRKCH |
| VDR | ESR2 | F2RL1 | SQLE | CYP2D6 | DRD3 | FNTA FNTB |
| PPARA | ATIC | TERT | DHFR | HTR6 | CTRC | EDNRB |
| CNR2 | PTPN22 | OPRK1 | CES2 | HTR1B | CYP2D6 | HTR2A |
| CDC25A | HDAC1 | IARS | ATP2A1 | POLB | HTR6 | MLNR |
| GLRA1 | MAP3K14 | HMGCR | FLT3 | FGF1 | HTR1B | CAPN1 |
| NR1H3 | MMP13 | TYMS | AURKA | GSK3B | FOLH1 | PPARG |
| MTNR1A | TLR9 | GART | TRPV4 | ADORA1 | POLB | ADRA1A |
| MTNR1B | ATP1A1 | PCSK7 | PGGT1B FNTA | BCL2L1 | FGF1 | OPRD1 |
| PTPN1 | PTGFR | PPM1A | PRKCG | RORC | GSK3B | SLC5A2 |
| ALOX5 | NPPA | FOLH1 | VAV1 | ATP1A1 | F2 | SLC5A1 |
| SLC6A2 | EDNRB | IKBKB | SERPINE1 | PPP1CC | HSD11B2 | F2RL1 |
| GC | ECE1 | TLR9 | NOS2 | IL2 | HSD11B1 | PDE4B |
| TOP2A | DLG4 | STAT3 | CTRC | FNTA FNTB | BCL2L1 | SLC33A1 |
| TNF | HMGCR | NOS2 | ADORA2A | LTB4R | GLI1 | IMPDH1 |
| PGR | PIK3CA | PDE4D | ADORA3 | PRKCD | JUN | IMPDH2 |
| FAAH | BACE1 | SLC46A1 | CNR1 | PRKCB | PRKCA | SLC10A2 |
| TRPV1 | AMPD3 | GSTM1 | CAPN1 | PRKCE | KCNA3 | HTR6 |
| CHRM2 | GLRA1 | PTPN1 | KDR | PRKCH | FDFT1 | ADRB2 |
| SLC6A4 | GLRA2 | TRPV4 | TTL | PRKCQ | STAT3 | ADRB1 |
| CYP2C19 | ADAMTS4 | SLC2A1 | CYP19A1 | PPM1B | PRKCD | ADRB3 |
| LTB4R | C3AR1 | ADCY1 | DNMT1 | PPP2CA | PRKCB | EGFR |
| NR1I3 | PSEN2 PSENEN NCSTN APH1A PSEN1 APH1B | SF3B3 | KDM4E | PPP2R5A | PRKCE | MMP13 |
| PTGS1 | ITGB1 ITGA5 | CXCR1 | KDM6B | MLNR | PRKCH | MMP3 |
| PPARG | HSD11B1 | PRKCG | KDM4A | PTPN22 | PRKCQ | MMP2 |
| PRKCA | HSD11B2 | VAV1 | KDM4C | KCNA3 | ATP2A1 | CTSB |
| RASGRP3 | NR3C1 | PTPN22 | IARS | STAT3 | PPM1B | MMP10 |
| NR3C2 | PTPN1 | PTAFR | PTGES | BCL2 | PTPN1 | MMP12 |
| NR3C1 | BCL2L1 | IL2 | ADRB2 | SERPINA6 | PPP1CC | MMP8 |
| SHBG | STAT3 | PLA2G1B | ADRB1 | F3 F7 | PPP2CA | IGF1R |
| CES2 | VDR | TRPV1 | PPARG | SELL | PPP2R5A | ITGA2B ITGB3 |
| BACE1 | MMP9 | SERPINA6 | PPARA | SELE | SQLE | ITGB5 ITGAV |
| SREBF2 | PTGFR | S1PR5 | PLA2G2A | NR3C2 | TERT | ITGAV ITGB1 |
| NPC1L1 | AR | S1PR4 | PCSK7 | PRKCG | VDR | HMGCR |
| CTSD | SERPINE1 | S1PR3 | SF3B3 | VAV1 | RORC | ADORA1 |
| CYP17A1 | PDE4A | LTB4R | CXCR1 | TRPV4 | ATP1A1 | HRH3 |
| HMGCR | PDE4B | S1PR1 | HRH1 | PTGS2 | PTGS2 | SIGMAR1 |
| CYP51A1 | GLI1 | IL1B | CCR3 | NOS2 | PTPA | ALDH2 |
| ALOX12 | THRA | SRC | GRB2 | MMP13 | GLRA1 | BACE1 |
| CES1 | THRB | DGAT1 | BCL2L1 | TERT | GLRA2 | SRD5A1 |
| MPI | MMP3 | PTGER1 | HSD11B2 | MMP12 | OPRM1 | ADORA2A |
| SRD5A1 | MMP8 | BCL2 | VDR | METAP2 | OPRK1 | ADORA3 |
| SRD5A2 | F2 | PDCD4 | GLI1 | F2RL1 | PDE4D | ALOX5AP |
| SERPINA6 | TYRO3 | LIPC | JUN | OPRM1 | F2RL1 | ITGAL |
| SIGMAR1 | JUN | LIPG | FDFT1 | PSEN2 PSENEN NCSTN APH1A PSEN1 APH1B | PPM1A | ITGA4 |
| NR1I2 | IL2 | GCG | HSD11B1 | ADRB2 | AR | ROCK2 |
| HSD17B3 | PDE5A | ADRA1A | AR | FGF1 | NR3C1 | S1PR3 |
| FABP1 | ITGAV ITGB3 | ADRA1B | MMP9 | SLC2A1 | MMP9 | S1PR1 |
| PTPN2 | MAPK14 | CCR6 | F2 | ADCY1 | KDR | SLC18A3 |
| HAO1 | MAPK10 | CNR2 | PPM1A | FOLH1 | HMGCR | BCL2L1 |
| PTPN6 | PTGER1 | PRSS3 | TLR9 | S1PR5 | TTL | HSD11B2 |
| LSS | TYMS | ATP6AP1 | HMGCR | S1PR4 | PSEN2 PSENEN NCSTN APH1A PSEN1 APH1B | HSD11B1 |
| ACHE | PTGER3 | FFAR1 | FFAR1 | S1PR3 | IKBKB | GLI1 |
| BCHE | PTGDR | STAT6 | GLRA1 | S1PR1 | FNTA FNTB | JUN |
| STAT3 | ITGA2B ITGB3 | NR3C2 | GLRA2 | AOC3 | PGGT1B FNTA | VDR |
| PTAFR | PTPN22 | MMP9 | FNTA FNTB | SQLE | PTAFR | PPM1A |
| IL2 | GRM2 | TPH1 | NR3C1 | AKT1 | NOS2 | GLRA1 |
| VEGFA | KDR | ABCB1 | SELL | HTR2B | IARS | GLRA2 |
| FGF1 | HSD17B1 | LTA4H | SELE | ADRA2A | SLC2A1 | F2 |
| FGF2 | CYP1A2 | CYP19A1 | RORC | ADRA2C | ADCY1 | AR |
| HPSE | CYP2C9 | METAP2 | ATP1A1 | ADRA2B | TLR9 | MMP9 |
| ATP1A1 | MMP12 | MLNR | PTPA | DRD1 | OPRD1 | PTAFR |
| PSEN2 PSENEN NCSTN APH1A PSEN1 APH1B | CASP1 | POLB | PTAFR | DRD2 | PRKCG | PTPN1 |
| RORC | FDFT1 | CASP3 | IL2 | ADRA1D | VAV1 | PTPA |
| HSP90AA1 | ADRB2 | SELL | KDR | HTR2A | TRPV4 | ITGB1 ITGA4 |
| LGALS4 | ADRB1 | SELE | PSEN2 PSENEN NCSTN APH1A PSEN1 APH1B | HTR2C | PCSK7 | TYMS |
| LGALS3 | FNTA FNTB | SELP | STAT3 | ADRA1A | GSTM1 | PTPRC |
| LGALS8 | NR1H4 | ITGA2B ITGB3 | PTPN1 | CYP2D6 | PLA2G1B | BACE1 |
| BCL2L1 | ITGB5 ITGAV | AKT1 | LTB4R | HTR6 | TRPV1 | TLR9 |
| CDK1 | ITGAV ITGB6 | ITGA2 ITGB3 | GART | HTR1B | SF3B3 | TBXAS1 |
| HSD11B2 | EDNRB | BCL2L1 | PPM1B | GART | NR3C2 | OPRK1 |
| HSD11B1 | GCK | HSD11B2 | PPP2CA | MMP9 | ABCB1 | ADORA3 |
| KDR | ELANE | GLI1 | PPP2R5A | ATP2A1 | GCGR | XIAP |
| HTR2B | CTSS | JUN | ALDH2 | DHODH | ADRA1A | IL2 |
| ADRA2A | DPP4 | FDFT1 | ITGA2B ITGB3 | PDE5A | ADRA1B | METAP2 |
| ADRA2C | BACE1 | PTPN1 | PRKCD | ITGA2B ITGB3 | CCR6 | OPRM1 |
| ADRA2B | PTGDR2 | PPM1A | PRKCB | LTA4H | MLNR | STAT3 |
| DRD1 | KMT5A | HSD11B1 | PRKCE | MMP2 | CXCR1 | AGTR2 |
| DRD2 | ITGAV ITGB1 | GLRA1 | PRKCH | GCG | SERPINA6 | KCNA3 |
| ADRA1D | HTR2B | GLRA2 | PRKCQ | PTPN2 | IL2 | PRKCD |
| HTR2A | HTR2C | TLR9 | SERPINA6 | DHFR | PDCD4 | PRKCB |
| HTR2C | CYP2D6 | RORC | NR3C2 | TPH1 | EDNRA | PRKCE |
| ADRA1A | HTR6 | ATP1A1 | TYMS | ITGA2 ITGB3 | METAP2 | PRKCH |
| DRD3 | HTR1B | PTAFR | DHFR | PFKFB3 | PLA2G2A | PRKCQ |
| CYP2D6 | MMP1 | F2 | METAP2 | BACE1 | ATP6AP1 | ADRB2 |
| HTR6 | BCL2 | PTPA | MLNR | PTGER1 | GSK3B | HLA-A |
| HTR1B | SELL | VDR | PTGS2 | CTRC | CYP19A1 | FNTA FNTB |
| PPM1B | SELP | ITGAV ITGB3 | F2RL1 | SLC46A1 | IL1B | FGF1 |
| PPP1CC | HMGCR | METAP2 | KCNA3 | FOLR1 | P2RY10 | SERPINA6 |
| PPP2CA | LIPC | TYMS | PPARA | SLC19A1 | GPR174 | ITGAV ITGB3 |
| PPP2R5A | CASP3 | ITGA2B ITGB3 | PPARD | HSD11B2 | PTPN2 | ITGB5 ITGAV |
| DCK | LIPG | PRKCD | FGF1 | HSD11B1 | PRSS1 | ITGAV ITGB6 |
| F2 | DGAT1 | PRKCB | PRKCG | F2 | CTRC | MMP13 |
| AR | ACE | PRKCE | VAV1 | GLI1 | FGF1 | NR3C2 |
| NR3C1 | TBXAS1 | PRKCH | TRPV4 | JUN | CA2 | PTPRA |
| MMP9 | RARG | PRKCQ | NOS2 | FDFT1 | CA7 | ITGA2B ITGB3 |
| TYMS | RARB | DHFR | TERT | VDR | CA1 | PTPN22 |
| BACE1 | RARA | SERPINA6 | HTR2B | NR3C1 | CA6 | HTR2B |
| ADRB2 | HRH1 | NR3C1 | ADRA2A | PTPN1 | CA12 | ADRA2A |
| LIPC | CCKBR | DGAT1 | ADRA2C | PPM1A | CA14 | ADRA2C |
| F10 | CCR3 | FOLH1 | ADRA2B | AR | CA9 | ADRA2B |
| LIPG | ITGB1 ITGA4 | HTR2B | DRD1 | GLRA1 | CA4 | DRD1 |
| HDAC6 | PSEN2 PSENEN NCSTN APH1A PSEN1 APH1B | ADRA2A | DRD2 | GLRA2 | CA13 | DRD2 |
| LNPEP | PPM1A | ADRA2C | ADRA1D | OPRK1 | CA5B | ADRA1D |
| HDAC8 | ADRB3 | ADRA2B | HTR2A | TLR9 | CA5A | HTR2A |
| HDAC1 | PIK3CA PIK3R1 | DRD1 | HTR2C | HMGCR | FKBP1A | HTR2C |
| IKBKB | PIK3CD | ADRA1D | DRD3 | PTPA | CNR2 | ADRA1A |
| MMP3 | PIK3CG | HTR2A | CYP2D6 | PTAFR | KCNH2 | DRD3 |
| MMP1 | PTAFR | HTR2C | HTR6 | BCL2L1 | RPS6KA5 | CYP2D6 |
| MMP2 | ANPEP | ADRA1A | HTR1B | HSD11B2 | FKBP4 | HTR6 |
| PTPRA | LNPEP | CYP2D6 | KDM4C | GLI1 | HSD11B1 | HTR1B |
| ANPEP | REN | HTR6 | PTGER1 | JUN | HSD11B2 | TERT |
| REN | FLT1 | HTR1B | SLC2A1 | FDFT1 | MMP9 | MLNR |
| OPRD1 | GRB2 | MLNR | ADCY1 | PTPN1 | PTPN1 | PTGS2 |
| XIAP | CTSA | PTGS2 | HSD17B1 | PPM1A | STAT3 | F2RL1 |
| ITGB1 ITGA4 | SLC22A6 | F2RL1 | CYP2C9 | HSD11B1 | BCL2L1 | HMGCR |
| TRPV1 | AKT2 | STAT3 | SQLE | GLRA1 | NR3C1 | LCK |
| METAP2 | MMP13 | KCNA3 | PTPN22 | GLRA2 | F2 | PTGDR2 |
| HLA-A | AKT1 | PSEN2 PSENEN NCSTN APH1A PSEN1 APH1B | PDE5A | TLR9 | GLI1 | ANPEP |
| S1PR1 | ITGB1 ITGA5 | FNTA FNTB | EPHA2 | RORC | IL2 | P2RY12 |
| ALDH2 | F10 | PTPN22 | SERPINE1 | ATP1A1 | FDFT1 | LNPEP |
| CASP3 | MMP2 | GART | CTRC | PTAFR | FFAR1 | CCKBR |
| GLI1 | ACP1 | TRPV4 | ITGA2 ITGB3 | F2 | VDR | F10 |
| PTPA | P2RY12 | F10 | CES2 | PTPA | PDE5A | CTSD |
| RRM1 | RBP4 | FGF1 | PTGES | VDR | SYK | PPP1CC |
| CDK9 CCNT1 | ADORA1 | SLC2A1 | ATP2A1 | ITGAV ITGB3 | CASP3 | AGTR1 |
| DYRK1A | ADORA2A | ADCY1 | PLG | METAP2 | CASP1 | FDFT1 |
| DYRK2 | ADORA3 | NPC1L1 | F10 | TYMS | PDE4A | GRB2 |
| DYRK3 | AR | PRKCG | PRSS3 | ITGA2B ITGB3 | PDE4B | SQLE |
| DYRK1B | CYP19A1 | VAV1 | SIRT3 | PRKCD | TYMS | DPP4 |
| CLK1 | ESR1 | SLC46A1 | SIRT1 | PRKCB | PTGER1 | PLK1 |
| CLK2 | ESR2 | ADRB2 | SLC6A2 | PRKCE | GRB2 | TACR3 |
| CLK3 | RORC | FOLR1 | TACR2 | PRKCH | SELL | ESR1 |
| CLK4 | HTR2B | MMP13 | SLC6A3 | PRKCQ | SELE | ESR2 |
| PRKCA | RXRB | TERT | ITGAV ITGB3 | DHFR | GART | ADORA2A |
| SLC6A2 | ADRA2B | TMPRSS15 | SLC6A4 | SERPINA6 | MMP13 | PTPN2 |
| TLR9 | RARG | TPH1 | SLC10A2 | NR3C1 | MMP2 | ATP2A1 |
| FANCF | RXRG | CES2 | TBXA2R | DGAT1 | MMP10 | LTB4R |
| S1PR3 | RARB | LTB4R | PGGT1B FNTA | FOLH1 | MMP12 | CASP1 |
| JUN | RARA | BCL2 | HSD11B2 | HTR2B | MMP8 | ABCB1 |
| FASN | RXRA | SELL | HSD11B1 | ADRA2A | RORC | PGGT1B FNTA |
| ADORA1 | MAPK14 | SELE | BCL2L1 | ADRA2C | IMPDH1 | CTSE |
| PRSS3 | RORB | DHODH | JUN | ADRA2B | IMPDH2 | SERPINE1 |
| PRSS1 | MAPK1 | ADAMTS4 | GLI1 | DRD1 | F10 | SLC2A1 |
| PFKFB3 | RORA | ITGB5 ITGAV | STAT3 | ADRA1D | ITGA2B ITGB3 | ADCY1 |
| ADORA2A | CHKA | ITGAV ITGB6 | KCNA3 | HTR2A | SERPINE1 | PRKCG |
| ADORA3 | VDR | SQLE | FDFT1 | HTR2C | PTAFR | VAV1 |
| S1PR5 | PPARA | GRB2 | PRKCD | ADRA1A | BACE1 | TRPV4 |
| S1PR4 | CNR2 | GCG | PRKCB | CYP2D6 | ACE | PDE5A |
| IGF1R | CDC25A | S1PR5 | PRKCE | HTR6 | MME | EGLN1 |
| ST14 | GLRA1 | ATIC | PRKCH | HTR1B | PTGDR2 | ACLY |
| EDNRB | NR1H3 | S1PR4 | PRKCQ | MLNR | ALDH2 | CHRNA7 |
| CTSD | MTNR1A | S1PR3 | RORC | PTGS2 | TYR | FDFT1 |
| ITGA2B ITGB3 | MTNR1B | S1PR1 | ATP1A1 | F2RL1 | ITGAV ITGB3 | HDAC3 |
| NPC1L1 | PTPN1 | BACE1 | VDR | STAT3 | PLG | AKR1B1 |
| NR1H3 | ALOX5 | CYP19A1 | PPM1B | KCNA3 | ITGB1 ITGA4 | HMGCR |
| RORC | SLC6A2 | TTL | PPP1CC | PSEN2 PSENEN NCSTN APH1A PSEN1 APH1B | ITGA4 | SLC1A1 |
| ESR1 | GC | TBXAS1 | PPP2CA | FNTA FNTB | PRSS3 | SLC1A2 |
| ESR2 | TOP2A | FFAR1 | PPP2R5A | PTPN22 | AR | HAO1 |
| SHBG | TNF | ATP2A1 | F2 | GART | DHFR | KDM2A |
| SREBF2 | PGR | CASP3 | PTPA | TRPV4 | ITGB7 ITGA4 | PHF8 |
| HMGCR | FAAH | FPGS | ATP2A1 | F10 | DUSP3 | KDM5C |
| CYP19A1 | TRPV1 | PRSS3 | SQLE | FGF1 | NRP1 | PTGER2 |
| AR | CHRM2 | MMP9 | TERT | SLC2A1 | PPP2CA | PTGFR |
| CYP17A1 | SLC6A4 | CTRC | PTPN1 | ADCY1 | HTR2B | FNTA FNTB |
| CYP51A1 | CYP2C19 | IARS | PTGS2 | NPC1L1 | ADRA2A | NPC1L1 |
| RORA | LTB4R | ITGB1 ITGA5 | NR3C1 | PRKCG | ADRA2C | UGT2B7 |
| CYP2C19 | NR1I3 | PTGDR2 | GLRA1 | VAV1 | ADRA2B | SLC6A1 |
| PTPN1 | PTGS1 | PTPN2 | GLRA2 | SLC46A1 | DRD1 | GABRA1 GABRB2 GABRG2 |
| SERPINA6 | PPARG | HMGCR | F2RL1 | ADRB2 | ADRA1D | GABRA3 GABRB2 GABRG2 |
| G6PD | PRKCA | BCL2L1 | TTL | FOLR1 | HTR2C | GABRA2 GABRB2 GABRG2 |
| ACHE | RASGRP3 | RORC | LTB4R | MMP13 | ADRA1A | GABBR2 GABBR1 |
| SLC6A4 | NR3C2 | ATP1A1 | NOS2 | TERT | DRD3 | GABRR1 |
| NR1I3 | NR3C1 | PPP1CC | PDE4D | TMPRSS15 | CYP2D6 | SLC6A11 |
| CHRM2 | SHBG | IL2 | F3 F7 | TPH1 | HTR6 | SLC22A6 |
| SLC6A2 | CES2 | FNTA FNTB | PPM1A | CES2 | HTR1B | SLC6A13 |
| BCHE | BACE1 | LTB4R | TPH1 | LTB4R | HSD17B1 | CPT1A |
| NR1H2 | SREBF2 | PRKCD | PRKCA | CYP1A2 | NOS2 | G6PD |
| PTGER1 | NPC1L1 | PRKCB | TLR9 | CYP2C9 | PLA2G1B | FABP4 |
| PTGER2 | CTSD | PRKCE | BCL2 | SRC | TRPV1 | PPARA |
| TBXAS1 | CYP17A1 | PRKCH | PRKCG | XIAP | OPRD1 | FABP3 |
| PTGES | HMGCR | PRKCQ | VAV1 | CTSA | TRPV4 | FABP5 |
| PPARA | CYP51A1 | PPM1B | TRPV4 | PIK3CA | SF3B3 | PPARD |
| PPARD | ALOX12 | PPP2CA | FFAR1 | LIPC | CXCR1 | FFAR1 |
| SQLE | CES1 | PPP2R5A | PCSK7 | LIPG | PRKCG | FABP2 |
| VDR | MPI | MLNR | SELL | MAP3K14 | VAV1 | ADRA2A |
| DHCR7 | SRD5A1 | PTPN22 | SELE | NPPA | IL2 | ADRA2C |
| PTPN6 | SRD5A2 | KCNA3 | LIPC | CASP6 | METAP2 | ADRA2B |
| PTPN2 | SERPINA6 | STAT3 | LIPG | CASP7 | EDNRA | DRD1 |
| FDFT1 | SIGMAR1 | BCL2 | ADRB2 | CASP8 | ATP1A1 | PRSS1 |
| HSD11B1 | NR1I2 | SERPINA6 | ADRB1 | ECE1 | GCGR | DRD2 |
| NOS2 | HSD17B3 | F3 F7 | MMP9 | PTGFR | ADRA1A | ADRA1D |
| PPARG | FABP1 | SELL | ALDH2 | AMPD2 | ADRA1B | HTR2A |
| UGT2B7 | PTPN2 | SELE | SLC2A1 | PPM1A | CCR6 | HTR2C |
| GLRA1 | HAO1 | NR3C2 | ADCY1 | CMA1 | NR3C2 | DRD3 |
| POLB | PTPN6 | PRKCG | SF3B3 | HDAC6 | ABCB1 | CTRC |
| PREP | LSS | VAV1 | TYMS | ESR1 | DHFR | CYP2D6 |
| PTGER4 | ACHE | TRPV4 | ITGA2B ITGB3 | ITGB5 ITGAV | SERPINA6 | HTR6 |
| IDO1 | BCHE | PTGS2 | PTAFR | ESR2 | IL1B | HTR1B |
| SIGMAR1 | JUN | NOS2 | FNTA FNTB | HDAC1 | PTPN2 | POLB |
| CES2 | GLI1 | MMP13 | GSTM1 | PPARG | PDCD4 | TLR9 |
| ATP12A | BCL2L1 | TERT | HTR4 | REN | P2RY10 | S1PR5 |
| PTGIR | KCNA3 | MMP12 | F10 | ITGAL | GPR174 | HSD11B2 |
| PDE4A | F2 | METAP2 | HMGCR | F3 F7 | MLNR | HSD11B1 |
| PDE4B | FDFT1 | F2RL1 | PLA2G1B | APP | PLA2G2A | BCL2L1 |
| PDE4C | PRKCD | OPRM1 | S1PR3 | EDNRB | ATP6AP1 |  |
| DHCR7 EBP | PRKCB | PSEN2 PSENEN NCSTN APH1A PSEN1 APH1B | S1PR1 | PPARA | CYP19A1 |  |
| FABP4 | PRKCH | ADRB2 | GIPR | PTPN22 | CNR2 |  |
| TERT | SLC2A1 | FGF1 | SSTR5 | MMP1 | HTR2B |  |

**Table s2: C**ommon targets between the disease and compound target datasets

| Names | total | elements | Target | 51 | GABRA3 GABRB2 GABRG2 |
| --- | --- | --- | --- | --- | --- |
| Genes Target | 397 | MMP2 |  |  | ITGAV ITGB1 |
|  |  | GRM2 |  |  | FNTA FNTB |
|  |  | CDC25B |  |  | TACR2 |
|  |  | PDCD4 |  |  | P2RY10 |
|  |  | SELL |  |  | CA5B |
|  |  | GRB2 |  |  | CA14 |
|  |  | DRD3 |  |  | PGGT1B FNTA |
|  |  | FKBP4 |  |  | SLC1A1 |
|  |  | CMA1 |  |  | ITGB7 ITGA4 |
|  |  | SQLE |  |  | CA13 |
|  |  | ALOX12 |  |  | FANCF |
|  |  | HTR4 |  |  | LPAR6 |
|  |  | LPAR3 |  |  | SLC6A11 |
|  |  | PTGER2 |  |  | CDK9 CCNT1 |
|  |  | ALOX5AP |  |  | DHCR7 EBP |
|  |  | MDM2 |  |  | GPR174 |
|  |  | AKT2 |  |  | CA12 |
|  |  | EGFR |  |  | PIK3CA PIK3R1 |
|  |  | KCNA3 |  |  | GLRA2 |
|  |  | F10 |  |  | ITGA2 ITGB3 |
|  |  | HRH1 |  |  | DYRK2 |
|  |  | RASGRP3 |  |  | CLK1 |
|  |  | PGR |  |  | FPGS |
|  |  | PLG |  |  | ITGA2B ITGB3 |
|  |  | ADRB3 |  |  | ITGB1 ITGA4 |
|  |  | HDAC1 |  |  | IARS |
|  |  | FABP2 |  |  | S1PR4 |
|  |  | SLC6A2 |  |  | CLK4 |
|  |  | MMP13 |  |  | ITGAV ITGB6 |
|  |  | AKR1B10 |  |  | TTL |
|  |  | MAP3K14 |  |  | PSEN2 PSENEN NCSTN APH1A PSEN1 APH1B |
|  |  | CLK2 |  |  | GABRA1 GABRB2 GABRG2 |
|  |  | ABCB1 |  |  | CASP6 |
|  |  | MAPK14 |  |  | PPP2R5A |
|  |  | DLG4 |  |  | F3 F7 |
|  |  | PLA2G4A |  |  | ITGB1 ITGA5 |
|  |  | FFAR1 |  |  | ITGAL ICAM1 ITGB2 |
|  |  | ACHE |  |  | SF3B3 |
|  |  | CASP3 |  |  | ITGAV ITGB3 |
|  |  | CA7 |  |  | LPAR5 |
|  |  | PRKCE |  |  | LPAR4 |
|  |  | PTPN1 |  |  | KDM2A |
|  |  | HTR2B |  |  | ITGB5 ITGAV |
|  |  | PGD |  |  | KDM4E |
|  |  | ITPR3 |  |  | ST14 |
|  |  | CYP2C19 |  |  | GABRA2 GABRB2 GABRG2 |
|  |  | ALDH2 |  |  | METAP1 |
|  |  | SRD5A2 |  |  | PPP1CC |
|  |  | NR1I3 |  |  | GABBR2 GABBR1 |
|  |  | NPPA |  |  | LGALS8 |
|  |  | CTSB |  |  |  |
|  |  | TNF |  |  |  |
|  |  | SELP |  |  |  |
|  |  | GLI1 |  |  |  |
|  |  | FDFT1 |  |  |  |
|  |  | PTGS2 |  |  |  |
|  |  | PIM3 |  |  |  |
|  |  | PDE4D |  |  |  |
|  |  | SLC19A1 |  |  |  |
|  |  | PIK3CD |  |  |  |
|  |  | PTGIR |  |  |  |
|  |  | PTPRC |  |  |  |
|  |  | DYRK3 |  |  |  |
|  |  | CCR6 |  |  |  |
|  |  | MAPK1 |  |  |  |
|  |  | HSD17B3 |  |  |  |
|  |  | CTRC |  |  |  |
|  |  | IKBKB |  |  |  |
|  |  | SRD5A1 |  |  |  |
|  |  | LIPG |  |  |  |
|  |  | HDAC8 |  |  |  |
|  |  | PDE4B |  |  |  |
|  |  | IMPDH1 |  |  |  |
|  |  | DCK |  |  |  |
|  |  | SLC6A4 |  |  |  |
|  |  | EPHA2 |  |  |  |
|  |  | PLA2G2A |  |  |  |
|  |  | FOLH1 |  |  |  |
|  |  | ADORA2A |  |  |  |
|  |  | PPARG |  |  |  |
|  |  | NR3C1 |  |  |  |
|  |  | CDK6 |  |  |  |
|  |  | LCK |  |  |  |
|  |  | TYRO3 |  |  |  |
|  |  | CHKA |  |  |  |
|  |  | RARA |  |  |  |
|  |  | ADRA2B |  |  |  |
|  |  | NPC1L1 |  |  |  |
|  |  | CA2 |  |  |  |
|  |  | CYP51A1 |  |  |  |
|  |  | ANPEP |  |  |  |
|  |  | CHRM2 |  |  |  |
|  |  | RRM1 |  |  |  |
|  |  | PRKCA |  |  |  |
|  |  | TOP1 |  |  |  |
|  |  | PTPN22 |  |  |  |
|  |  | CYP2D6 |  |  |  |
|  |  | ROCK2 |  |  |  |
|  |  | F2RL1 |  |  |  |
|  |  | BCHE |  |  |  |
|  |  | BCL2 |  |  |  |
|  |  | F7 |  |  |  |
|  |  | MAPK10 |  |  |  |
|  |  | CCR3 |  |  |  |
|  |  | PTPN2 |  |  |  |
|  |  | CYP19A1 |  |  |  |
|  |  | MMP1 |  |  |  |
|  |  | TRPV1 |  |  |  |
|  |  | MMP3 |  |  |  |
|  |  | DUSP3 |  |  |  |
|  |  | PTPN6 |  |  |  |
|  |  | PPP2CA |  |  |  |
|  |  | TYR |  |  |  |
|  |  | LIPC |  |  |  |
|  |  | AMPD2 |  |  |  |
|  |  | PTGER3 |  |  |  |
|  |  | HTR2C |  |  |  |
|  |  | FOLR1 |  |  |  |
|  |  | TBXA2R |  |  |  |
|  |  | PRKCD |  |  |  |
|  |  | CNR1 |  |  |  |
|  |  | SLC1A2 |  |  |  |
|  |  | ADAMTS4 |  |  |  |
|  |  | ITPR1 |  |  |  |
|  |  | CASP7 |  |  |  |
|  |  | GART |  |  |  |
|  |  | SCARB1 |  |  |  |
|  |  | PTPA |  |  |  |
|  |  | SIGMAR1 |  |  |  |
|  |  | LPAR2 |  |  |  |
|  |  | VDR |  |  |  |
|  |  | ACE |  |  |  |
|  |  | CYP1A2 |  |  |  |
|  |  | PIK3CG |  |  |  |
|  |  | S1PR1 |  |  |  |
|  |  | KDM4C |  |  |  |
|  |  | SYK |  |  |  |
|  |  | GABRR1 |  |  |  |
|  |  | SIRT3 |  |  |  |
|  |  | TLR9 |  |  |  |
|  |  | AKT1 |  |  |  |
|  |  | PLA2G4B |  |  |  |
|  |  | LPAR1 |  |  |  |
|  |  | JUN |  |  |  |
|  |  | CA6 |  |  |  |
|  |  | AR |  |  |  |
|  |  | INPP5A |  |  |  |
|  |  | APP |  |  |  |
|  |  | SLC33A1 |  |  |  |
|  |  | S1PR2 |  |  |  |
|  |  | SMO |  |  |  |
|  |  | FABP5 |  |  |  |
|  |  | SLC22A6 |  |  |  |
|  |  | RARB |  |  |  |
|  |  | ECE1 |  |  |  |
|  |  | GC |  |  |  |
|  |  | KIT |  |  |  |
|  |  | CA5A |  |  |  |
|  |  | MVD |  |  |  |
|  |  | SREBF2 |  |  |  |
|  |  | ENPP2 |  |  |  |
|  |  | ADRA1D |  |  |  |
|  |  | SLC2A1 |  |  |  |
|  |  | HMGCR |  |  |  |
|  |  | FLT1 |  |  |  |
|  |  | GSTM1 |  |  |  |
|  |  | ICMT |  |  |  |
|  |  | CTSE |  |  |  |
|  |  | PHF8 |  |  |  |
|  |  | IL1B |  |  |  |
|  |  | REN |  |  |  |
|  |  | CTSA |  |  |  |
|  |  | MMP10 |  |  |  |
|  |  | PDE4A |  |  |  |
|  |  | ADRA2C |  |  |  |
|  |  | FASN |  |  |  |
|  |  | NR1H3 |  |  |  |
|  |  | PTPN11 |  |  |  |
|  |  | DPP4 |  |  |  |
|  |  | AOC3 |  |  |  |
|  |  | TMPRSS15 |  |  |  |
|  |  | LTB4R |  |  |  |
|  |  | ITGA4 |  |  |  |
|  |  | KDR |  |  |  |
|  |  | RORA |  |  |  |
|  |  | BACE1 |  |  |  |
|  |  | RBP4 |  |  |  |
|  |  | PPM1B |  |  |  |
|  |  | ADORA1 |  |  |  |
|  |  | IL2 |  |  |  |
|  |  | SLC46A1 |  |  |  |
|  |  | PTAFR |  |  |  |
|  |  | SLC5A1 |  |  |  |
|  |  | SLC5A2 |  |  |  |
|  |  | PTGS1 |  |  |  |
|  |  | CA1 |  |  |  |
|  |  | ATIC |  |  |  |
|  |  | MMP9 |  |  |  |
|  |  | RPS6KA5 |  |  |  |
|  |  | GPR55 |  |  |  |
|  |  | CDK1 |  |  |  |
|  |  | PPM1A |  |  |  |
|  |  | CAPN1 |  |  |  |
|  |  | GNRHR |  |  |  |
|  |  | ADRA1A |  |  |  |
|  |  | GIPR |  |  |  |
|  |  | HDAC3 |  |  |  |
|  |  | HCRTR1 |  |  |  |
|  |  | CASP1 |  |  |  |
|  |  | KMT5A |  |  |  |
|  |  | TYMS |  |  |  |
|  |  | MME |  |  |  |
|  |  | AMPD3 |  |  |  |
|  |  | LGALS3 |  |  |  |
|  |  | G6PD |  |  |  |
|  |  | THRA |  |  |  |
|  |  | ACP1 |  |  |  |
|  |  | CSNK2A1 |  |  |  |
|  |  | LNPEP |  |  |  |
|  |  | PTGDR |  |  |  |
|  |  | PTGFR |  |  |  |
|  |  | SELE |  |  |  |
|  |  | FAAH |  |  |  |
|  |  | MPI |  |  |  |
|  |  | HLA-A |  |  |  |
|  |  | EDNRB |  |  |  |
|  |  | STAT3 |  |  |  |
|  |  | TPH1 |  |  |  |
|  |  | SOAT1 |  |  |  |
|  |  | POLB |  |  |  |
|  |  | LSS |  |  |  |
|  |  | CNR2 |  |  |  |
|  |  | HRH3 |  |  |  |
|  |  | CCR1 |  |  |  |
|  |  | HTR6 |  |  |  |
|  |  | FGF2 |  |  |  |
|  |  | RARG |  |  |  |
|  |  | FABP4 |  |  |  |
|  |  | HSD11B1 |  |  |  |
|  |  | SSTR5 |  |  |  |
|  |  | MDM4 |  |  |  |
|  |  | RXRB |  |  |  |
|  |  | HSD17B1 |  |  |  |
|  |  | HSD11B2 |  |  |  |
|  |  | CES2 |  |  |  |
|  |  | EDNRA |  |  |  |
|  |  | PTGES |  |  |  |
|  |  | OPRK1 |  |  |  |
|  |  | DHFR |  |  |  |
|  |  | ATP1A1 |  |  |  |
|  |  | CYP2C9 |  |  |  |
|  |  | ESR1 |  |  |  |
|  |  | CDK4 |  |  |  |
|  |  | DHCR7 |  |  |  |
|  |  | MMP12 |  |  |  |
|  |  | GLRA1 |  |  |  |
|  |  | CXCR3 |  |  |  |
|  |  | AKR1B1 |  |  |  |
|  |  | SRC |  |  |  |
|  |  | CYP17A1 |  |  |  |
|  |  | PRKCQ |  |  |  |
|  |  | PRKCH |  |  |  |
|  |  | IGF2R |  |  |  |
|  |  | PCSK7 |  |  |  |
|  |  | S1PR3 |  |  |  |
|  |  | DRD1 |  |  |  |
|  |  | NRP1 |  |  |  |
|  |  | KDM5C |  |  |  |
|  |  | KCNH2 |  |  |  |
|  |  | PIM1 |  |  |  |
|  |  | F11 |  |  |  |
|  |  | ATP2A1 |  |  |  |
|  |  | RORB |  |  |  |
|  |  | CTSS |  |  |  |
|  |  | PDPK1 |  |  |  |
|  |  | TBXAS1 |  |  |  |
|  |  | PFKFB3 |  |  |  |
|  |  | AGTR1 |  |  |  |
|  |  | CCKBR |  |  |  |
|  |  | ADRB2 |  |  |  |
|  |  | PRSS3 |  |  |  |
|  |  | CTSD |  |  |  |
|  |  | SLC10A2 |  |  |  |
|  |  | PTGDR2 |  |  |  |
|  |  | HSP90AA1 |  |  |  |
|  |  | RXRG |  |  |  |
|  |  | FABP1 |  |  |  |
|  |  | FLT3 |  |  |  |
|  |  | BCL2L1 |  |  |  |
|  |  | MAPK3 |  |  |  |
|  |  | ADK |  |  |  |
|  |  | NR1H4 |  |  |  |
|  |  | OPRM1 |  |  |  |
|  |  | SERPINE1 |  |  |  |
|  |  | DYRK1A |  |  |  |
|  |  | ATP12A |  |  |  |
|  |  | S1PR5 |  |  |  |
|  |  | HDAC6 |  |  |  |
|  |  | NOS2 |  |  |  |
|  |  | PRKCG |  |  |  |
|  |  | PRKCB |  |  |  |
|  |  | ELANE |  |  |  |
|  |  | UGT2B7 |  |  |  |
|  |  | SHBG |  |  |  |
|  |  | PREP |  |  |  |
|  |  | HCRTR2 |  |  |  |
|  |  | THRB |  |  |  |
|  |  | FGF1 |  |  |  |
|  |  | PTGER1 |  |  |  |
|  |  | SHH |  |  |  |
|  |  | XIAP |  |  |  |
|  |  | CA9 |  |  |  |
|  |  | LGALS4 |  |  |  |
|  |  | CLK3 |  |  |  |
|  |  | GCGR |  |  |  |
|  |  | KDM6B |  |  |  |
|  |  | ACLY |  |  |  |
|  |  | VEGFA |  |  |  |
|  |  | GSK3B |  |  |  |
|  |  | ADCY1 |  |  |  |
|  |  | PPARD |  |  |  |
|  |  | KDM4A |  |  |  |
|  |  | IDO1 |  |  |  |
|  |  | CXCR1 |  |  |  |
|  |  | MMP8 |  |  |  |
|  |  | SLC6A1 |  |  |  |
|  |  | PDE5A |  |  |  |
|  |  | METAP2 |  |  |  |
|  |  | TACR3 |  |  |  |
|  |  | HPSE |  |  |  |
|  |  | CPT1A |  |  |  |
|  |  | AKT3 |  |  |  |
|  |  | ITGAL |  |  |  |
|  |  | CHRNA7 |  |  |  |
|  |  | CDC25A |  |  |  |
|  |  | RXRA |  |  |  |
|  |  | SLC18A3 |  |  |  |
|  |  | FABP3 |  |  |  |
|  |  | EGLN1 |  |  |  |
|  |  | ATP6AP1 |  |  |  |
|  |  | NR1H2 |  |  |  |
|  |  | STAT6 |  |  |  |
|  |  | MTNR1A |  |  |  |
|  |  | PIK3CA |  |  |  |
|  |  | OPRD1 |  |  |  |
|  |  | TOP2A |  |  |  |
|  |  | IGF1R |  |  |  |
|  |  | HIF1A |  |  |  |
|  |  | NR1I2 |  |  |  |
|  |  | CSF1R |  |  |  |
|  |  | CES1 |  |  |  |
|  |  | PRSS1 |  |  |  |
|  |  | MTNR1B |  |  |  |
|  |  | IMPDH2 |  |  |  |
|  |  | GCK |  |  |  |
|  |  | ADRA2A |  |  |  |
|  |  | PDE4C |  |  |  |
|  |  | AURKA |  |  |  |
|  |  | ADRB1 |  |  |  |
|  |  | DGAT1 |  |  |  |
|  |  | F2 |  |  |  |
|  |  | PTGER4 |  |  |  |
|  |  | NR3C2 |  |  |  |
|  |  | CA4 |  |  |  |
|  |  | PLA2G1B |  |  |  |
|  |  | PTPRF |  |  |  |
|  |  | TRPV4 |  |  |  |
|  |  | ADORA3 |  |  |  |
|  |  | TERT |  |  |  |
|  |  | RORC |  |  |  |
|  |  | SIRT1 |  |  |  |
|  |  | HTR2A |  |  |  |
|  |  | ESR2 |  |  |  |
|  |  | AGTR2 |  |  |  |
|  |  | SLC6A3 |  |  |  |
|  |  | LTA4H |  |  |  |
|  |  | SERPINA6 |  |  |  |
|  |  | ALOX5 |  |  |  |
|  |  | PLK1 |  |  |  |
|  |  | C3AR1 |  |  |  |
|  |  | HAO1 |  |  |  |
|  |  | CASP8 |  |  |  |
|  |  | MTOR |  |  |  |
|  |  | DRD2 |  |  |  |
|  |  | ADRA1B |  |  |  |
|  |  | P2RY12 |  |  |  |
|  |  | SLC6A13 |  |  |  |
|  |  | DYRK1B |  |  |  |
|  |  | PPARA |  |  |  |
|  |  | HTR1B |  |  |  |
|  |  | DNMT1 |  |  |  |
|  |  | PTPRA |  |  |  |
|  |  | GCG |  |  |  |
|  |  | MLNR |  |  |  |
|  |  | DHODH |  |  |  |
|  |  | FKBP1A |  |  |  |
|  |  | VAV1 |  |  |  |

**Table s3:** Degree of freedom of Top 10 gene

| Top 10 in network Sheet1 ranked by Degree method |  |  |
| --- | --- | --- |
| Rank | Name | Score |
| 1 | AKT1 | 184 |
| 2 | TNF | 175 |
| 3 | SRC | 167 |
| 4 | IL1B | 147 |
| 5 | EGFR | 138 |
| 6 | STAT3 | 134 |
| 7 | PPARG | 123 |
| 8 | CASP3 | 122 |
| 9 | MAPK3 | 118 |
| 10 | BCL2 | 116 |

**Table s4:**2D interaction of Gymnema Sylvestre containing bioactive constituents with AKT1

| Gymnemaside VI  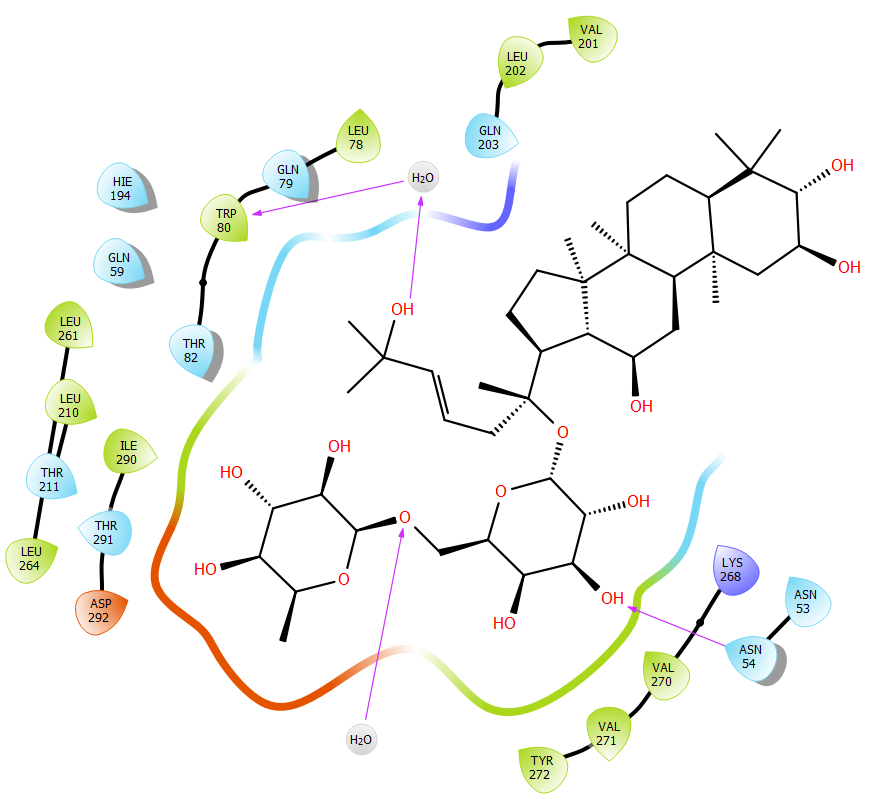 | Phytic acid  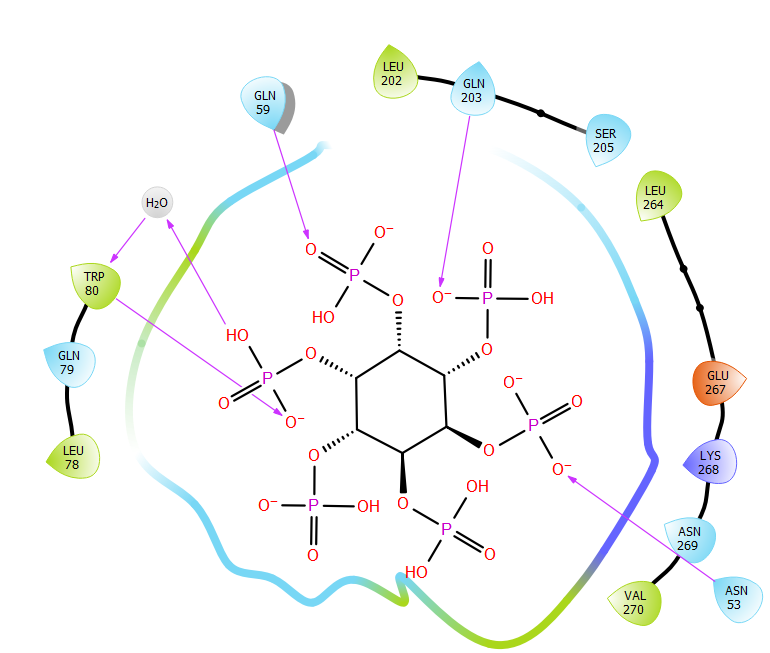 |
| --- | --- |
| Gymnemic acid II  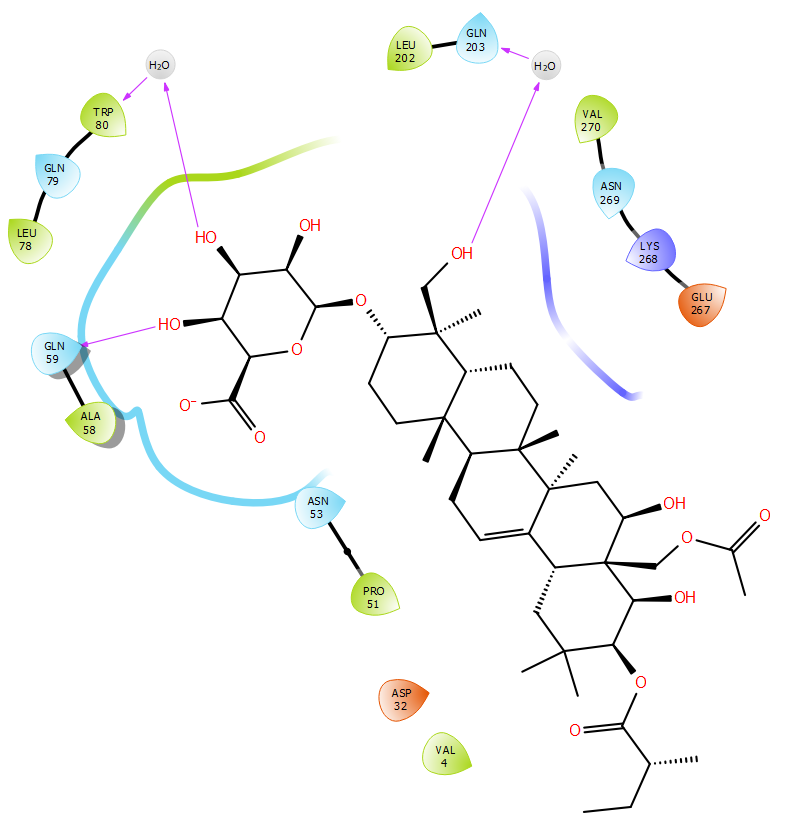 | Deacylgymnemic acid  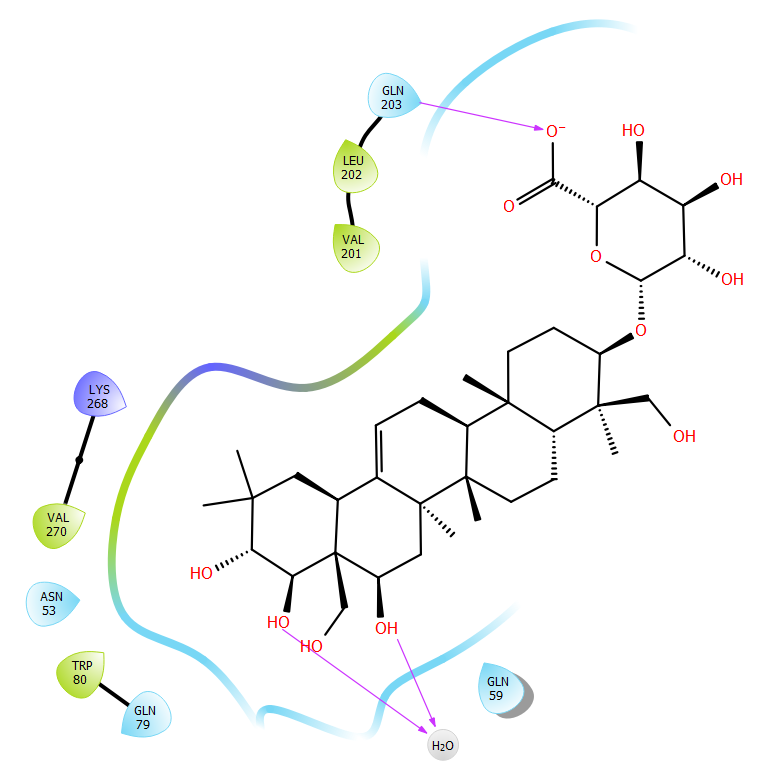 |
| Gymnemic acid IV  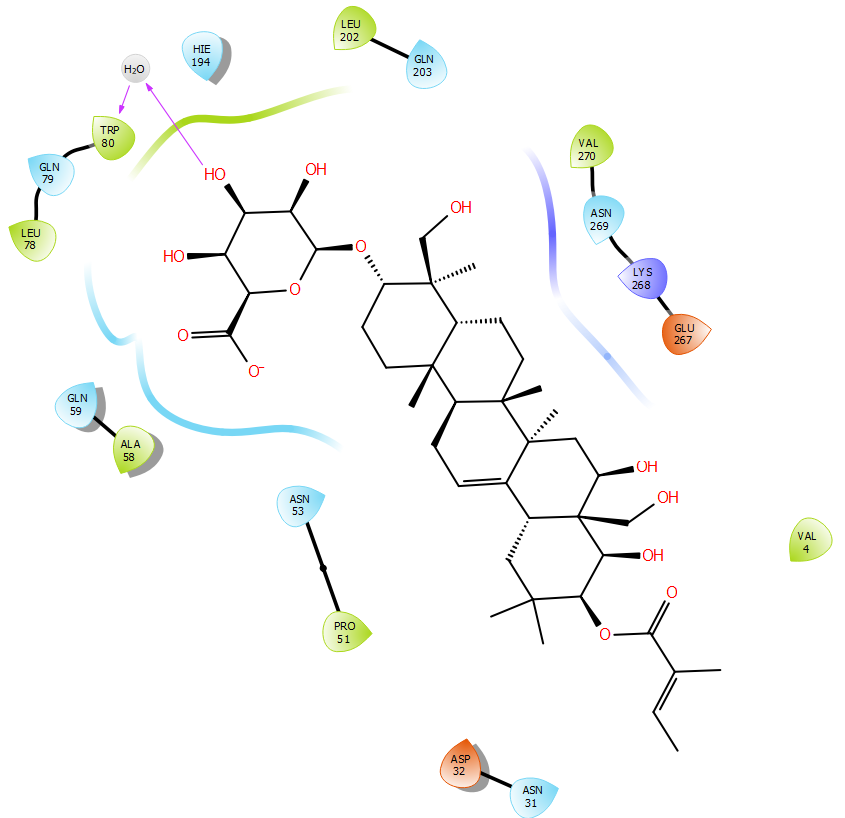 | Stigmasterol  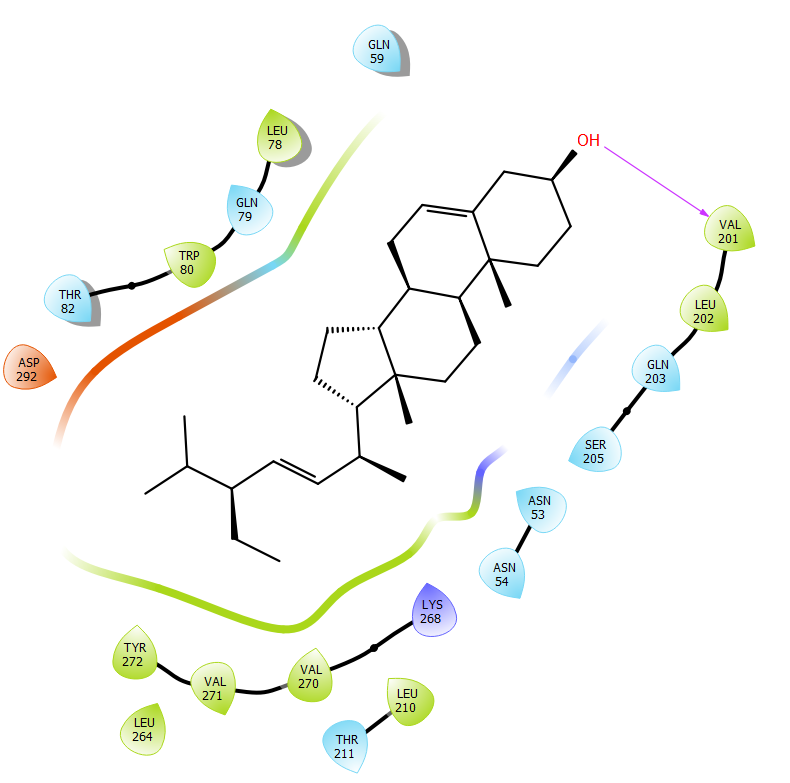 |
| Longispinogenin 3-O-beta-D-glucuronopyranoside  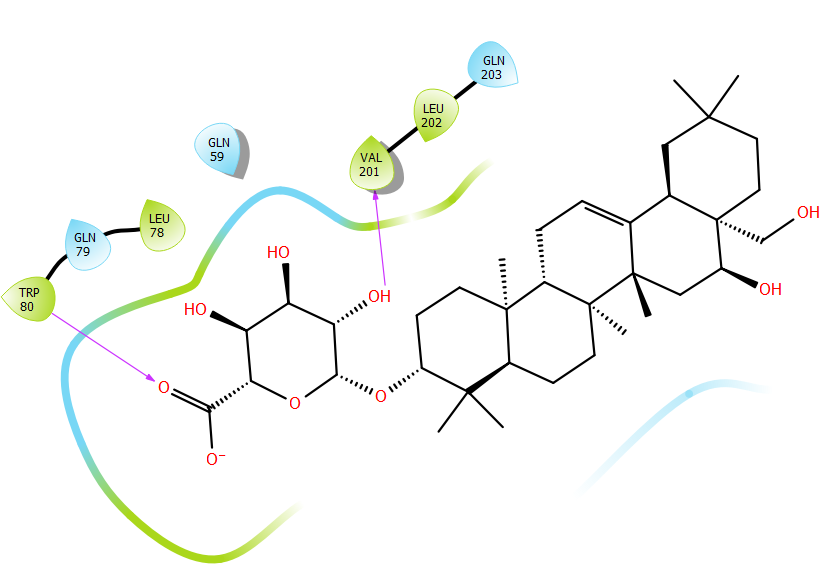 | Gymnemic acid X  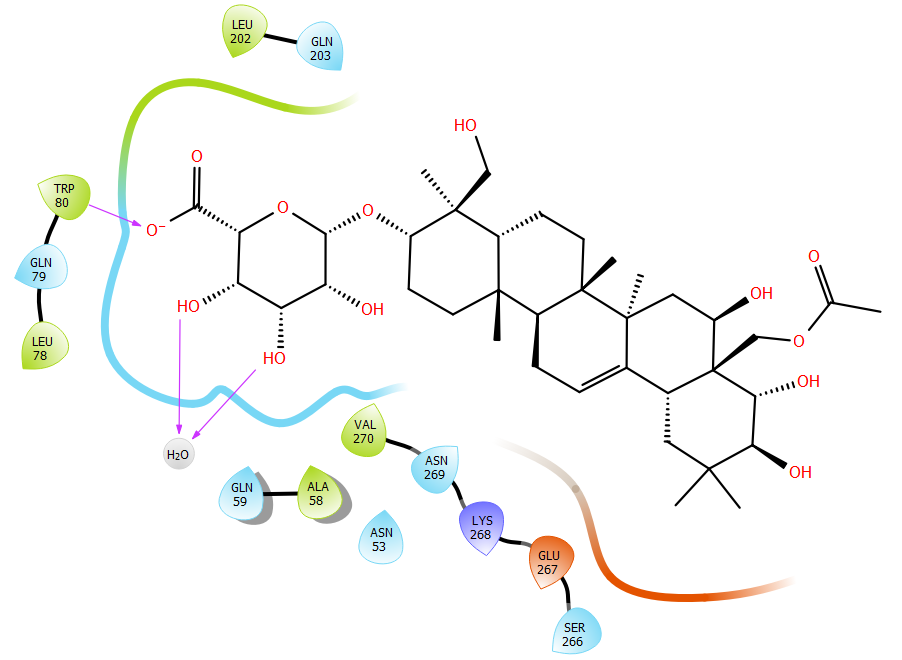 |
| beta-Amyrin acetate  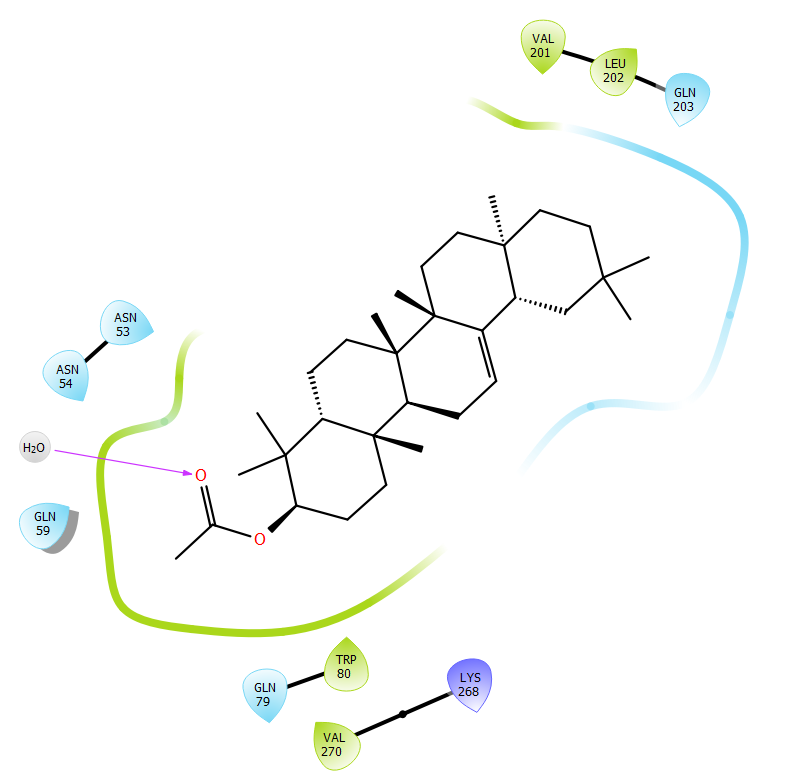 |  |
|  |  |
|  |  |
|  |  |
|  |  |
|  |  |

**Table s5:** Graph showing the radius of gyration, the solvent-accessible surface area (SASA) and other parameters of a protein-ligand complex.

| **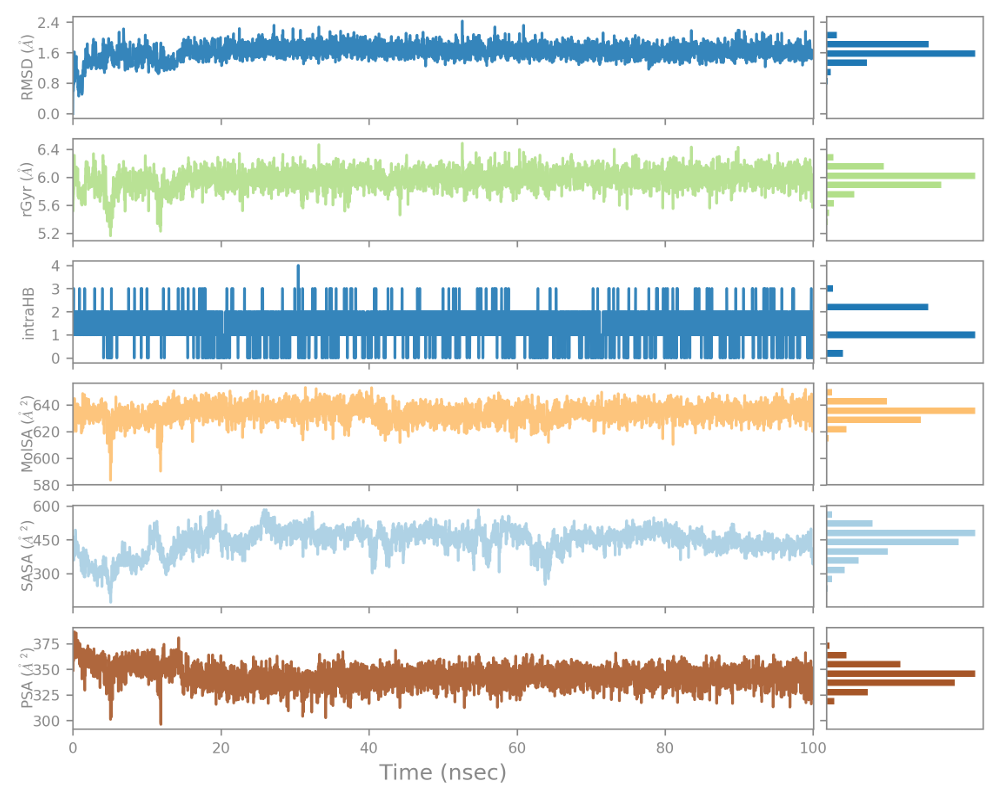** |
| --- |
